# Impact of Peptide Sequence on Functional siRNA Delivery and Gene Knockdown with Cyclic Amphipathic Peptide Delivery Agents

**DOI:** 10.1021/acs.molpharmaceut.3c00455

**Published:** 2023-11-14

**Authors:** Melissa L. Jagrosse, Uday K. Baliga, Christopher W. Jones, Jade J. Russell, Claudia I. García, Rauf Ahmad Najar, Arshad Rahman, David A. Dean, Bradley L. Nilsson

**Affiliations:** † Department of Chemistry, University of Rochester, Rochester, New York 14627-0216, United States; ‡ Materials Science Program, University of Rochester, Rochester, New York 14627, United States; § Department of Pediatrics and Neonatology, University of Rochester Medical Center, School of Medicine and Dentistry, University of Rochester, Rochester, New York 14642, United States

**Keywords:** drug delivery, siRNA delivery, cell-penetrating peptide, cyclic peptide

## Abstract

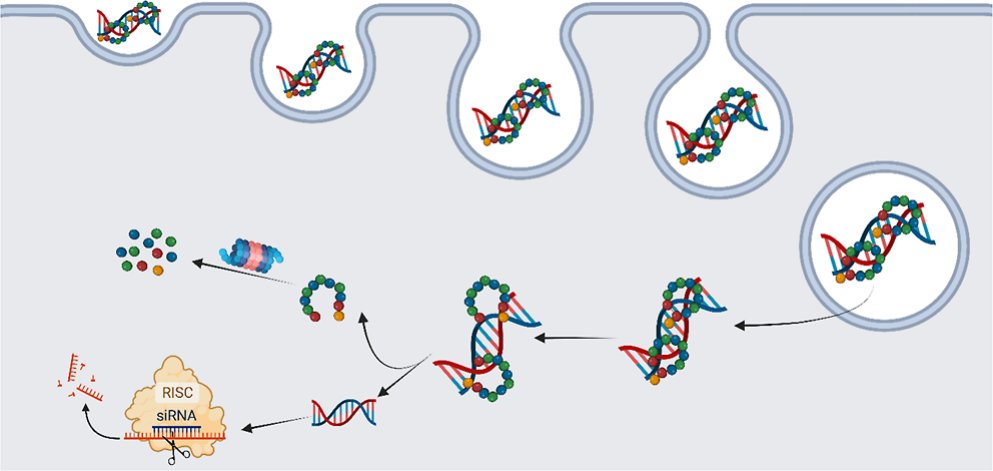

Short-interfering RNA (siRNA) oligonucleotide therapeutics that modify gene expression by accessing RNA-interference (RNAi) pathways have great promise for the treatment of a range of disorders; however, their application in clinical settings has been limited by significant challenges in cellular delivery. Herein, we report a structure–function study using a series of modified cyclic amphipathic cell-penetrating peptides (CAPs) to determine the impact of peptide sequence on (1) siRNA-binding efficiency, (2) cellular delivery and knockdown efficiency, and (3) the endocytic uptake mechanism. Nine cyclic peptides of the general sequence Ac-C[XZ]_4_CG-NH_2_ in which X residues are hydrophobic/aromatic (Phe, Tyr, Trp, or Leu) and Z residues are charged/hydrophilic (Arg, Lys, Ser, or Glu) are assessed along with one acyclic peptide, Ac-(WR)_4_G-NH_2_. Cyclization is enforced by intramolecular disulfide bond formation between the flanking Cys residues. Binding analyses indicate that strong cationic character and the presence of aromatic residues that are competent to participate in CH–π interactions lead to CAP sequences that most effectively interact with siRNA. CAP–siRNA binding increases in the following order as a function of CAP hydrophobic/aromatic content: His < Phe < Tyr < Trp. Both cationic charge and disulfide-constrained cyclization of CAPs improve uptake of siRNA *in vitro*. Net neutral CAPs and an acyclic peptide demonstrate less-efficient siRNA translocation compared to the cyclic, cationic CAPs tested. All CAPs tested facilitated efficient siRNA target gene knockdown of at least 50% (as effective as a lipofectamine control), with the best CAPs enabling >80% knockdown. Significantly, gene knockdown efficiency does not strongly correlate with CAP–siRNA internalization efficiency but moderately correlates with CAP–siRNA-binding affinity. Finally, utilization of small-molecule inhibitors and targeted knockdown of essential endocytic pathway proteins indicate that most CAP–siRNA nanoparticles facilitate siRNA delivery through clathrin- and caveolin-mediated endocytosis. These results provide insight into the design principles for CAPs to facilitate siRNA delivery and the mechanisms by which these peptides translocate siRNA into cells. These studies also demonstrate the nature of the relationships between peptide–siRNA binding, cellular delivery of siRNA cargo, and functional gene knockdown. Strong correlations between these properties are not always observed, which illustrates the complexity in the design of optimal next-generation materials for oligonucleotide delivery.

## Introduction

Many diseases are caused by aberrant protein expression or the expression of protein variants with an altered function. The ability to modify gene expression by the administration of exogenous agents thus has great potential for the treatment of these types of disorders. First discovered in *Caenorhabditis elegans* in 1998,^1^ RNA interference (RNAi) is an endogenous pathway that induces gene silencing by utilizing information stored in exogenous or endogenous double-stranded RNAs (dsRNAs).^1−8^ These exogenous or endogenous dsRNA molecules are termed short-interfering RNA (siRNA). The cellular RNAi machinery utilizes siRNA to induce gene silencing with high target specificity and rapid, efficient, and short-term degradation of mRNA(s).^1−6,8^ Exogenous dsRNA is processed into double-stranded siRNA by the endoribonuclease Dicer.^9^ The siRNA–Dicer complex is then loaded into the RNA-induced silencing complex, where the double-stranded siRNA is unwound, the passenger strand is degraded, and the remaining guide strand is used for target mRNA recognition through complementary binding.^6^ Upon sequence recognition, the argonaute-2 protein (Ago2) binds the complementary mRNA to inhibit translational machinery or to signal for degradation, depending on siRNA–mRNA mismatching.^3,6^

RNAi-based therapeutics provide promising avenues for personalized medicine in a variety of diseases and disorders, including but not limited to, metabolic disorders,^10^ viral infections,^11^ cancer,^12,13^ and bone regeneration.^14^ The considerable promise of exploiting RNAi pathways for therapeutic applications has been impeded by barriers to cytosolic delivery of siRNA oligonucleotides.^15−19^ The major challenge for the development of RNAi-based therapeutics requires the development of delivery vectors that facilitate siRNA packaging, cell binding, internalization, and unpackaging of siRNA cargo and ultimate presentation of the siRNA duplex to the RNAi machinery. Numerous siRNA delivery systems have been explored, including viral vectors,^20−25^ lipofection,^5,26^ cationic polymers,^27−30^ antibody constructs,^31^ and RNA aptamers.^32,33^ Although significant progress in RNAi delivery has been made with the aforementioned transfection agents, none are ideal, and significant barriers have thus far limited clinical applications with these agents.^34−38^

Cell-penetrating peptides (CPPs) are promising transfection agents for siRNA delivery.^39−43^ A wide range of CPPs have been exploited, with some of the most common being transportan,^44^ VP22,^45^ MAP,^46^ synthetic arginine-rich peptides,^47,48^ and TAT.^7,49^ Specifically, peptides rich in tryptophan (W) and arginine (R) residues have been shown to effectively deliver siRNA *in vitro*. Modified linear peptides are one promising class of peptides that have been shown to self-assemble into nanostructures, such as micelles, to facilitate siRNA delivery.^50^ Montazeri Aliabadi et al. demonstrated efficient cellular uptake (50–90%) in a cell line that is traditionally difficult to transfect (MDA-MB-231 triple-negative cancer cells) using linear peptides containing tryptophan and arginine repeats separated by β-alanine spacers.^50^ This efficient cellular uptake translated to superior knockdown (<90%) of the signal transducer and activator of transcription 3 (STAT3) in SKOV-3 cells.^50^

**Figure 1:**
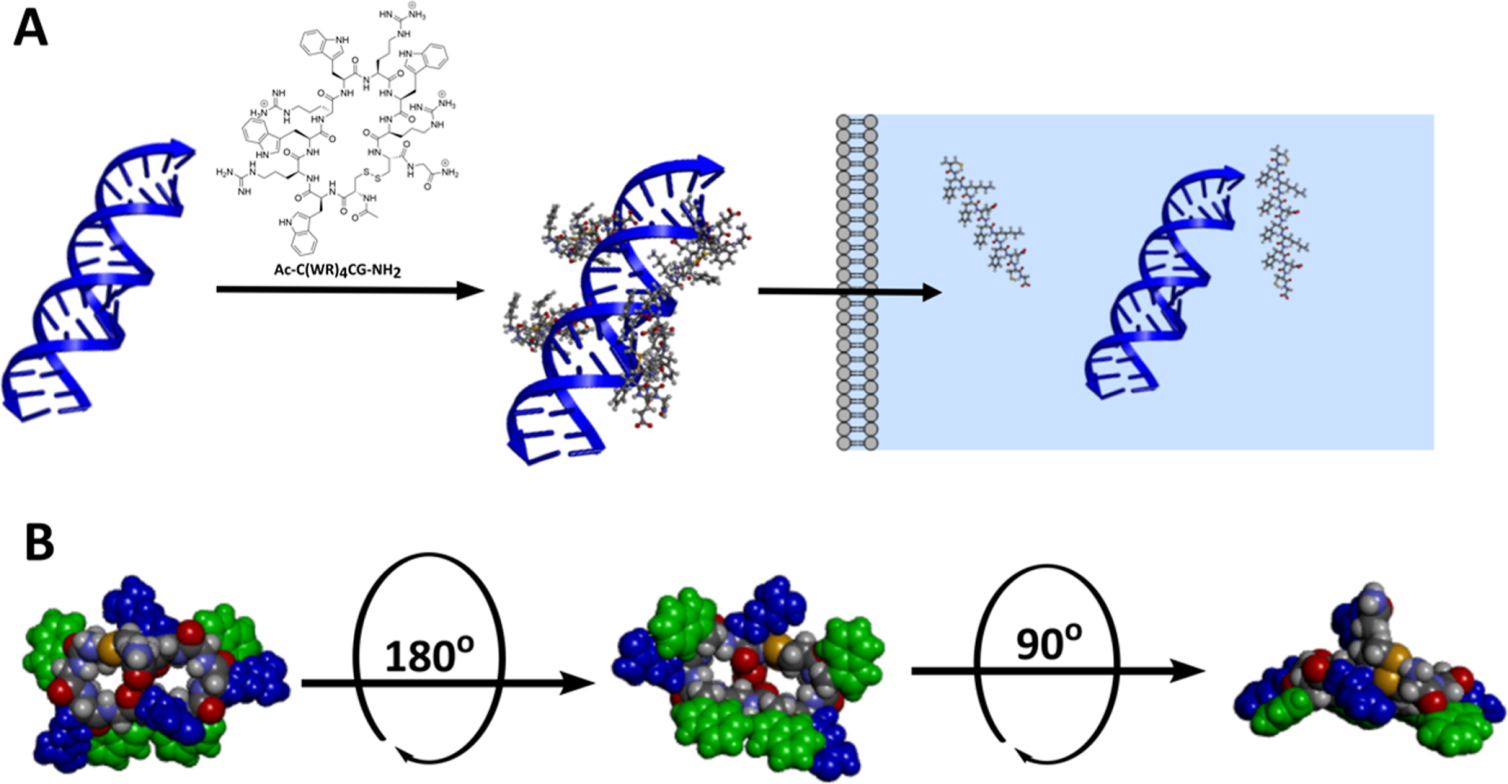
(A) Graphical representation of condensation of disulfide-constrained CAPs with siRNA into CAP siRNA nanoparticles that facilitate translocation of siRNA across cell membranes into the cytosol.^55^ (B) Structural representation of the cyclic-Ac-C[WR]_4_G-NH_2_ CAP in which the indole side chain groups of Trp residues are shown in green, the Arg guanidium-presenting side chains are shown in blue, and the Cys sulfur residues forming the constraining disulfide bond are shown in yellow.

We and others have reported cyclic amphipathic CAPs for the functional cytosolic delivery of siRNA (Figure 1A).^51−55^ Montazeri Aliabadi et al. evaluated the cellular uptake and knockdown efficiency of cyclic [FR]_4_, [WR]_5_, and [WK]_4_ peptides in the presence and absence of a lipid delivery vector (1,2-dioleoysl-*sn*-glycero-3-phosphoethanolamine, DOPE) in triple-negative breast cancer cell lines.^56^ Although [FR]_4_ and [WK]_5_ demonstrated negligible siRNA delivery, the authors showed that the addition of DOPE significantly increased internalization of siRNA with all three cyclic peptides.^56^ Similarly, codelivery of [WR]_5_ with DOPE generated knockdown efficiency on-par with commercial lipofectamine.^56^ We have previously reported CAPs consisting of alternating hydrophobic and hydrophilic residues that are flanked by cysteines at each terminus (Figure 1B), which then undergo oxidative intramolecular disulfide bond formation to affect cyclization of the peptide.^55^ CAPs condense with siRNA to form peptide/oligonucleotide nanoparticles that facilitate the translocation of complexed siRNA into the cytosol of cells. In the reducing environment of the cytosol, the constraining disulfide bond undergoes reduction with cytosolic glutathione^57^ and the linearized peptides are degraded by cellular proteases, thus enabling presentation of the siRNA cargo to the RNAi machinery. We found that CAPs enriched in aromatic Trp and cationic Arg residues (cyclic-Ac-C[WR]_4_CG-NH_2_) had dramatically superior delivery and knockdown properties both *in vitro* and *in vivo* compared to CAPs that were net neutral and with Phe residues in the aromatic positions (cyclic-Ac-C[FKFE]_2_CG-NH_2_).^55^ The cyclic-Ac-C[WR]_4_CG-NH_2_ CAP affected over 90% reduction of thyroid transcription factor-1 (TTF-1) expression *in vitro* and in the lung in *in vivo* mouse models without noticeable off-target effects.^55^ Additionally, we investigated the knockdown efficiency of a cyclic [WR]_4_ variant that was cyclized via thioether bond formation and was not sensitive to reductive ring-opening. This variant demonstrated reduced knockdown (∼45%) of TTF-1 *in vitro* relative to the disulfide bonded cyclic peptide (>90% knockdown), suggesting that reductive ring-opening is essential for adequate unpacking of siRNA for availability to the cellular machinery.^55^

Based on these promising results, we have sought to further understand the physicochemical basis of CAP complexation with siRNA and the mechanisms of cellular internalization of CAP–siRNA complexes.^55^ The mode of CAP interaction with siRNA leading to condensation into CAP–siRNA nanoparticles is critical to understand to design next-generation CAPs. Previously reported data suggested that CAPs bind to siRNA most effectively when the hydrophilic amino acids are cationic and when the hydrophobic amino acids are aromatic. Does the nature of the cation (ammonium vs guanidinium) or aromatic group (benzene or indole) affect siRNA complexation? In addition, how do these functional CAP groups impact delivery of the siRNA cargo into cells and functional knockdown of the gene target? Cellular uptake of siRNA complexed with other CPPs has been shown to occur via endocytic or nonendocytic pathways, depending on the particle size, peptide type, and siRNA-loading method.^42,58^ Nanoparticles are commonly endocytosed by clathrin-coated pits, caveolae, or macropinocytosis,^59^ with less-common pathways including Cdc42, CLIC/GEEC pathways, and membrane ruffling creating transient nanopores.^60,61^ This complicates structure–function design choices that would account for the uptake mechanism(s) and could be used to improve delivery efficiency and targeting capabilities.^62^

Accordingly, we report herein the results of structure–function analyses designed to provide insight into how the CAP sequence influences both CAP–siRNA condensation and cytosolic delivery of siRNA and functional knockdown of gene targets. We designed and synthesized a series of CAPs in which both aromatic (Phe, Trp, Tyr, and His) and charged/hydrophilic residues (Lys, Arg, Glu, Ser, and His) were varied to enable comparative siRNA-binding affinity studies and characterization of the condensed nanoparticles. These studies provide insight into specific effects of amino acid functional groups on CAP interactions with siRNA. In addition, the efficiency of cytosolic siRNA delivery and gene target knockdown of the resulting CAP–siRNA particles was assessed to further elucidate the relationship between CAP sequence and functional siRNA delivery to the RNAi machinery. Finally, a comparative functional characterization of endocytic uptake mechanisms of these complexes was conducted to determine how the CAP sequence might impact the mode of cellular delivery. Collectively, these studies confirm that both aromatic and cationic characters influence CAP–siRNA-binding affinity and complex formation. CAP sequence also correlates with delivery and knockdown efficiency. Mechanistic internalization studies conclude that most CAP–siRNA nanoparticles primarily utilize clathrin- and/or caveolin-mediated translocation across the cell membrane, which can be correlated with CAP sequence and CAP–siRNA-binding affinity. Collectively, these studies delineate key design principles for the optimization of CAPs for the delivery of siRNA to cells.

## Materials and Methods

### Materials

All reagents and solvents were purchased from commercial vendors and used without further purification. Vendors for specific reagents are provided in the following sections when appropriate.

### Peptide Synthesis, Purification, and Characterization

Peptides (Figure S1, Supporting Information) were prepared by solid-phase peptide synthesis using previously reported methods.^55,63,64^ Peptides were synthesized using standard Fmoc-protection and HBTU activation/coupling protocols. Peptide cyclization was performed under oxidative conditions using 2,2′-dipyridyl disulfide (PDS).^55,63^ PDS (5 mL, 3.75 mM in MeOH) was added to peptide (90 μM) dissolved in 60% MeCN/H_2_O with stirring at room temperature for 1 h. Peptides were lyophilized, purified by high-performance liquid chromatography (HPLC) on an Interchim Puriflash 4125, and characterized on a Shimadzu Axima Performance matrix-assisted laser desorption ionization-time-of-flight (MALDI-TOF) mass spectrometer and Shimadzu LC-2010A analytical HPLC (see the Supporting Information for HPLC conditions and supporting data, including HPLC traces, concentration curves, and MALDI-MS spectra, Figures S2–S22 and Tables S1 and S2.)

### Slot Blot Filtration Assay^65^

Nitrocellulose (Bio-Rad 1620112) and nylon (VWR 95038-400) membranes were soaked in 100 mM Tris buffer for 20 min. The membranes were then stacked on top of two sheets of Bio-Rad SF thick filter paper (no. 1620161) in a Bio-Rad SF 48-well slot blot apparatus. Tris buffer (pH 7.5, 200 μL, 100 mM) was added to each well, and vacuum was applied to test for even vacuum distribution and membrane rehydration. This process was repeated three times. Peptides (0–150 μM) were dissolved in H_2_O (123.5 μL) and incubated with FITC–siRNA (Santa Cruz Biotechnology sc-36869, 6.5 μL, 1 μM) for 30 min. Samples (40 μL) were added to the wells with additional Tris buffer (pH 7.5, 160 μL) in triplicate and covered with aluminum foil for 20 min. The vacuum was then opened, allowing samples in each well to penetrate the membranes. After the vacuum was closed, 200 μL of Tris buffer was added to rinse each well. Tris buffer was added, and the vacuum was reapplied; this was repeated three times. Membranes were then imaged using a Bio-Rad ChemiDoc Touch. Fluorescence intensity density measurements were made using ImageJ. The fraction of siRNA bound to the nitrocellulose membrane was determined using eq 1:
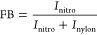
1where FB is the fraction bound, *I*_nitro_ is the fluorescence intensity measured from the nitrocellulose membrane, and *I*_nylon_ is the fluorescence intensity measured from the nylon membrane

CAP-binding affinities (*K*_d_) were extrapolated using eq 2:
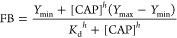
2in which*X* is the ligand (CAP) concentration (μM), *Y*_max_ is the maximal plateau of fraction bound, *Y*_min_ is the minimal plateau of fraction bound, and *h* is the Hill slope.

See the Supporting Information for nitrocellulose and nylon membrane images (Figures S23–S32) and plots of fraction bound [siRNA] vs [CAP] (Figure S33).

### Nitrogen/Phosphate Ratio

The peptide/siRNA ratio was calculated based on the nitrogen/phosphate (N/P) ratio using eq 3:
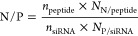
3where *n*_peptide_ is the number of moles of peptide, *n*_siRNA_ is the number of moles of siRNA, *N*_N/peptide_ is the number of protonatable nitrogens per peptide molecule, and *N*_P/siRNA_ is the number of phosphate groups per siRNA molecule.

### Dynamic Light Scattering

CAPs (50 or 0.5 μM) were mixed with TTF-1 siRNA (Santa Cruz Biotechnology sc-36756, 50 nM) and allowed to stand for 30 min at 25 °C. Measurements were taken using a DynaPro Plate Reader II. Radius is reported as an average of 10 measurements using Dynamics software. See the Supporting Information for plots of dynamic light scattering (DLS) data (Figures S34–S43) and a comparison of radii for the various siRNA–CAP nanoparticles (Figure S44).

### Transmission Electron Microscopy

CAPs (50 or 0.5 μM) were complexed with TTF-1 siRNA (Santa Cruz Biotechnology sc-36756, 50 nM) and allowed to stand for 30 min at 25 °C. Samples of these mixtures (5 μL) were spotted on 200 mesh carbon-coated copper grids and were allowed to stand for 60 s after which liquid was carefully removed by capillary action using filter paper. Grids were then stained with uranyl acetate (5 μL) for 60 s before removing excess stain by capillary action with filter paper. Grids were allowed to dry for 10 min. Images were taken by using a Hitachi 7650 transmission electron microscope with an accelerating voltage of 80 kV. ImageJ was used to analyze particle sizes captured on transmission electron microscopy (TEM). The spatial scale of the image was defined by drawing a straight-line selection across the image scale bar. This distance in pixels was used to calibrate the spatial scale under “Analyze/Set Scale”. Particle sizes are reported as the average of 20 measurements of unique particles, with the error reported as the standard deviation. See the Supporting Information for TEM images (Figures S34–S43).

### CAP–siRNA Nanoparticle Condensation for Cell-Based Assays

For microscopic visualization to determine the efficiency of siRNA internalization, CAP–siRNA complexes (132 nM siRNA and 13.3 μM CAP) were prepared by combining an aliquot of each CAP solution (40 nmol, 8 μL, 500 μM, H_2_O) with 40 pmol of siGlo Red solution [Dharmacon D-001630-02, 4 μL in phosphate-buffered saline (PBS) solution]. For functional knockdown experiments, CAP–siRNA complexes (33 nM siRNA and 33 μM CAP) were prepared by combining an aliquot of each CAP solution (10 nmol, 1 μL, 500 μM, H_2_O) with scrambled siRNA solution (Santa Cruz Biotechnology sc-37007, 10 pmol, 1 μL, 10 μM, PBS) or TTF-1 siRNA solution (Santa Cruz Biotechnology sc-36756, 10 pmol, 1 μL, 10 μM, PBS). Complexes were incubated at room temperature in the dark for 30 min and then diluted into 300 μL of Dulbecco’s modified Eagle medium (DMEM) without fetal bovine serum (FBS) or antibiotics with/without internalization inhibitors or dimethylsulfoxide (DMSO) as necessary.

### Microscopic siRNA Internalization Measurements

A549 alveolar epithelial cells (CCL-185, ATCC) were counted and plated onto glass coverslips grown in 12-well tissue culture plates until 70–90% confluent. Media was removed from the cells and replaced with CAP–siGlo complexes (300 μL, PBS described above) at 37 °C in 5% CO_2_ for 4 h. Cells were then rinsed with PBS (500 μL) and fixed for 10 min in PBS containing 4% paraformaldehyde. After fixation, coverslips used for CAP–siGlo uptake were stained with Wheat Germ Agglutinin-Alexa 488 (300 μL, 5 μg/mL in PBS) for 10 min to stain the cell membrane, rinsed three times with PBS (500 μL), stained with 4,6-diamidino2-phenylindole (DAPI) (300 μL, 5 μg/μL in PBS) for 5 min, rinsed three times with PBS (500 μL), mounted on slides with Prolong gold antifade mounting media (12 μL), and sealed with clear nail polish. Coverslips were observed under a Leica DMRXA2 epifluorescence microscope with a 20× objective (Leica, Wetzlar, Germany). Images were acquired with a Hamamatsu Ocra-ER 12 bit, cooled CCD camera (Hamamatsu, Japan) and Volocity imaging software. Captured images were then analyzed using ImageJ/FIJI to quantify the number of siGlo “spots” within a cell (based on wheat germ agglutinin outlining of cell membranes).

### TTF-1 mRNA Knockdown Measurements

A549 alveolar epithelial cells (CCL-185, ATCC) were grown to 70–90% confluency in 12-well plates and then washed with DMEM without FBS or antibiotics. CAP–TTF1–siRNA nanoparticle solution (300 μL, 33 nM siRNA, 33 μM CAP, DMEM without FBS or antibiotics) was then added to each well, and cells were incubated at 37 °C, 5% CO_2_ for 4 h. Media were then removed and replaced with complete media (10% FBS 1% Pen–Strep DMEM), and cells were incubated at 37 °C, 5% CO_2_ for an additional 48 h before RNA extraction using an Aurum Total RNA Mini Kit (Bio-Rad 7326820). In brief, cells were lysed with lysis solution containing 1% β-mercaptoethanol (200 μL). Lysate was then mixed with 70% EtOH (200 μL) and processed using a QIAshredder homogenizer column (Qiagen 79656). The homogenate was then transferred to Aurum RNA-binding columns, washed with low-stringency wash solution, and incubated with diluted DNase I solution (80 μL) for 15 min at RT. Samples were then serially washed with high- and low-stringency wash solutions, and RNA was eluded from the column with ultrapure DNase-/RNase-free distilled water (Invitrogen 10977015, 80 μL). Recovered RNA was quantified on a NanoDrop One system, and samples were diluted in ultrapure DNase-/RNase-free distilled water (Invitrogen 10977015, 2 ng/μL). Relative TTF-1 knockdown was quantified by reverse-transcription quadrupole polymerase chain reaction (RT-qPCR) (see following sections for details).

### Reverse-Transcription Quadrupole Polymerase Chain Reaction

An iScript cDNA Synthesis Kit (Bio-Rad 1708891) was used for reverse transcription of the extracted RNA samples. In brief, samples were prepared by combining 5× iScript reaction mix (4 μL), iScript reverse transcriptase (1 μL), extracted RNA (12.5 μL and 2 ng/μL), and ultrapure DNase-/RNase-free distilled water (Invitrogen 10977015, 2.5 μL). Samples were incubated on a Bio-Rad Thermal Cycler using the following protocol: 5 min priming at 25 °C, 20 min reverse transcription at 40 °C, and 1 min reverse-transcriptase inactivation at 95 °C. Recovered cDNA was quantified on a NanoDrop One, and samples were diluted in ultrapure DNase-/RNase-free distilled water (Invitrogen 10977015, 50 ng/μL). Gene expression was quantified by utilizing quadrupole polymerase chain reaction (qPCR). A primer stock solution (750 nM, 267 μL, H_2_O) of TTF-1 (Santa Cruz Biotechnology, sc-36756 PR) or GAPDH (Santa Cruz Biotechnology, sc-35448 PR) primer pairs were prepared by dilution of primer A (20 μL, 10 μM) and primer B (20 μL, 10 μM) into ultrapure DNase-/RNase-free distilled water (Invitrogen 10977015, 227 μL). Samples were prepared in triplicate by combining 2× iTaq Universal SYBR Green Super Mix (5 μL, Bio-Rad), TTF-1, or GAPDH primer stock (4 μL, 750 nM) and recovered cDNA (1 μL, 50 ng/μL) in sterile qPCR tubes. Quantitative *C*_*t*_ values were obtained by running samples on a Bio-Rad CFX96 Touch instrument using the “PWRUP60” protocol. Gene expression values were calculated by utilizing a variation of the Livak method, in which GAPDH was used as the reference gene.

### *In Vitro* Pathway Inhibition Experiments

A549 cells were counted and plated onto glass coverslips grown in 12-well tissue culture plates until they were 70–90% confluent. Media were removed, and cells were pretreated with an inhibitor or DMSO in DMEM without FBS or antibiotics for 1 h. Media were removed from the cells and incubated with CAP–siGlo solution (300 μL) with an inhibitor or DMSO at 37 °C in 5% CO_2_ for 4 h. Cells were then rinsed with PBS (500 μL) and fixed for 10 min in PBS containing 4% paraformaldehyde. For uptake controls, A594-holotransferrin (Invitrogen T13343, 16.7 μg/mL, H_2_O) was used as a marker of clathrin-mediated endocytosis and BODIPY-lactosyl ceramide (Invitrogen B22650, 0.81 μM, H_2_O) was used as a marker of caveolae-mediated endocytosis. Control markers (3.4 μL of A594-holotransferrin and 1.6 μL BODIPY-lactosyl ceramide) were added to 1 mL of DMEM without serum or antibiotics, and 300 μL of this solution was added to cells on coverslips, incubated at 37 °C 5% CO_2_ for 30 min before being rinsed with PBS (500 μL) and fixed for 10 min in PBS containing 4% paraformaldehyde. After fixation, coverslips used for CAP–siGlo uptake were stained with Wheat Germ Agglutinin-Alexa 488 (300 μL, 5 μg/mL in PBS) for 10 min to stain the cell membrane, rinsed three times with PBS (500 μL), stained with DAPI (300 μL, 5 μg/μL in PBS) for 5 min, rinsed three times with PBS (500 μL), mounted on slides with Prolong gold antifade mounting media (12 μL), and sealed with clear nail polish. Coverslips were observed under a Leica DMRXA2 epifluorescence microscope with a 20× or 63× objective (Leica, Wetzlar, Germany). Images were acquired with a Hamamatsu Ocra-ER 12 bit, cooled CCD camera (Hamamatsu, Japan), and Volocity imaging software. The inhibitors were Dynasore (DNS) (Abcam ab120192, 400 μM in DMSO) and Monodansylcadaverine (Sigma-Aldrich D4008, 200 μM in DMSO).

### *In Vitro* Pathway Knockdown Experiments

A549 cells were harvested, counted, and then resuspended at 2 × 10^6^ cells in 250 μL of electroporation buffer (120 mM KCl, 0.15 mM CaCl_2_, 10 mM K_2_HPO_4_, 10 mM KH_2_PO_4_, 25 mM HEPES, 2 mM EGTA, and 5 mM MgCl_2_). Cav1–siRNA (Santa Cruz Biotechnology sc-29241, 15 pmol, 1.5 μL of 10 μM) for caveolin-1 or CLTC–siRNA (Origene SR300867, 15 pmol, 1.5 μL of 10 μM) for Clathrin heavy chain was added, and these suspensions were then transferred to 0.2 cm electroporation cuvettes before a square-wave 250 V, 2000 μF, 1000 Ω, 20 ms, single pulse was applied using a Bio-Rad Gene Pulser MXCell. Cells were then recovered in DMEM containing 10% FBS plated at 5 × 10^5^ cells/well in a six-well plate for 24 h before uptake experiments and/or protein extraction by Reporter Lysis Buffer (Promega E397A) per manufacturer recommendations. Knockdown was analyzed via Western blot analysis, as described above.

### Western Blotting

Extracted protein (30 μL) was mixed with 4× loading dye [10 μL, 0.25 M Tris-HCl, pH 6.8, 40% glycerol, 8% sodium dodecyl sulfate (SDS), 0.04% bromophenol blue, and 20% β-mercaptoethanol] and then heated at 95 °C for 5 min before being placed on ice. Samples (15 μL) were run on 8% SDS-polyacrylamide gel electrophoresis gels, followed by transfer to a polyvinylidene fluoride (PVDF) membrane at 4 °C at constant 40 mA for 20 h. PVDF membranes were then washed with PBS-T three times, blocked in 5% milk PBS-T for 45 min, and incubated for 2 h with a primary antibody (blot-dependent, described below) in 5% milk PBS-T for 16 h at 4 °C. Membranes were then incubated with a horseradish peroxidase (HRP)-conjugated secondary antibody against the primary antibody host species (either antirabbit or antimouse, described below) in 5% milk PBS-T for 1 h at room temperature and visualized with the Clarity ECL Western blotting reagent (Bio-Rad, Hercules, CA) as per manufacturer recommendations using the ChemiDoc imaging system (Bio-Rad, Hercules, CA). Densitometry was performed using an Image Lab (Bio-Rad). Expression was normalized relative to that of GAPDH, which was used as a loading control. Primary antibodies were anti-Clathrin heavy chain (Abcam 172958) and were used at 1:10,000. Anti-Caveolin-1 (CST 3267) was used at 1:1000. Anti-GAPDH (Millipore Sigma MAB374) was used at 1:10,000. Anti-TTF1 (Abcam ab76013) was used at 1:2000. Secondary antibodies were Goat antirabbit HRP (Bio-Rad 170-6515, 1:5000) or Goat antimouse HRP (Pierce 1858413, 1:10,000).

### Cytotoxicity Assays^66^

Cytotoxicity was measured by LDH release using the Cyquant LDH Cytotoxicity assay (Invitrogen C20300). A549 cells were plated at 2 × 10^5^ cells/well of a 24-well plate and incubated for 24 h at 37 °C, 5% CO_2_. Cells were then washed with serum-free media, followed by incubation with CAP–siGlo (132 nM siRNA and 13.3 μM CAP) complexes in serum-free media for 4 or 24 h. At 4 h and again at 24 h, media (two 50 μL aliquots from each well) were removed from the cells and dispensed into 96-well plates for the assay. The LDH colorimetric substrate (50 μL) was added to the media aliquot and incubated for 30 min before the reaction was stopped (50 μL of STOP solution), and then the absorbance was read (680 nm for baseline subtraction and 490 nm for the colorimetric substrate) on a plate reader (Spectramax M2 using Softmax Pro). Media without cells were used for quantifying background absorbance. Lysed cells were used as a standard for 100% LDH release, and untreated cells were used for spontaneous LDH release. Cytotoxicity of CAP–siRNA complexes was compared to that of Lipofectamine 2000 via Ordinary one-way analysis of variance analysis.

## Results and Discussion

### CAP Design and Synthesis

A series of CAPs were designed to assess structure–function relationships between the constituent amino acids of the CAP and binding affinity to siRNA and the morphological properties of the resulting CAP–siRNA particles. These CAPs (Table 1) were of the general sequence cyclic-Ac-C[XZ]_4_CG-NH_2_ in which X residues are aromatic (Phe, Tyr, His, and Trp) or hydrophobic (Leu) amino acids and Z residues are hydrophilic/charged amino acids. Cyclization of these peptides was induced by intramolecular oxidative disulfide bond formation between the sulfur groups of the flanking Cys residues. CAP sequences were designed to enable the analysis of specific aromatic and charge effects on siRNA binding and condensation, cytosolic delivery, and functional knockdown. Aromatic residues (X) included Phe, Tyr, and Trp; a nonaromatic residue, Leu, was also included in the X position as a general hydrophobic control. Residues in the Z position included Lys and Glu (to give the net neutral [FKFE]_2_ CAP, Table 1), cationic Lys, Arg, or His residues, and neutral Ser residues. Nine CAP sequences with these amino acid variations allowed the correlation of CAP aromatic/hydrophobic content and charge structure to siRNA binding and delivery. We previously reported [FKFE]_2_ and [WR]_4_ for delivery of siRNA *in vitro* and wanted to analyze these peptides further in this study.^55^ In addition, a noncyclic, linear derivative, Ac-(WR)_4_G-NH_2_, an analogue of our previously reported [WR]_4_ CAP,^55^ was included to assess the effect of cyclization on peptide–siRNA interactions. We included the [FK]_4_ CAP to investigate the impact of the net neutral character of [FKFE]_2_ on the knockdown efficiency. Additionally, we wanted to investigate the impact of the cationic side chain (arginine vs lysine) and aromatic side chain (phenylalanine vs tyrosine vs tryptophan) in various combinations on the binding affinity, cellular internalization of siRNA, knockdown efficiency, and cellular uptake mechanism.

**Table 1:** CAP Sequences, Abbreviations, and Kinetic Data^a^

CAP sequence	abbreviation	apparent *K*_d_ (μM)	Hill slope (*h*)	CAP–siRNA particle cluster radius (nm)
cyclic-Ac-C[FKFE]_2_CG-NH_2_	[FKFE]_2_	29.0 ± 2.8	2.8 ± 0.5	188 ± 11
cyclic-Ac-C[FK]_4_CG-NH_2_	[FK]_4_	11.1 ± 0.2	16.4 ± 2.3	247 ± 35
cyclic-Ac-C[FR]_4_CG-NH_2_	[FR]_4_	14.0 ± 1.5	1.6 ± 0.3	212 ± 25
cyclic-Ac-C[LR]_4_CG-NH_2_	[LR]_4_	8.6 ± 0.2	4.9 ± 0.7	209 ± 25
cyclic-Ac-C[YR]_4_CG-NH_2_	[YR]_4_	3.1 ± 0.4	1.3 ± 0.1	135 ± 18
cyclic-Ac-C[WR]_4_CG-NH_2_	[WR]_4_	2.8 ± 0.3	2.1 ± 0.1	202 ± 20
cyclic-Ac-C[WK]_4_CG-NH_2_	[WK]_4_	0.87 ± 0.05	2.8 ± 0.5	135 ± 20
cyclic-Ac-C[WH]_4_CG-NH_2_	[WH]_4_	26.7 ± 3.3	2.2 ± 0.5	210 ± 30
cyclic-Ac-C[WS]_4_CG-NH_2_	[WS]_4_	26.6 ± 3.8	2.0 ± 0.2	291 ± 33
Ac-(WR)_4_G-NH_2_	(WR)_4_G	30.0 ± 1.1	2.7 ± 0.2	189 ± 9

a Slot blot filtration assays were utilized to obtain apparent dissociation constants (*K*_d_) and Hill slope values (*h*). Values are reported as the average of at least three measurements, with the error reported as the standard deviation. Radii of CAP–siRNA nanoparticles were measured via DLS and are reported as the average of at least three measurements, with the error reported as the standard deviation.

All peptides were synthesized using standard solid-phase peptide synthesis with Fmoc protection protocols.^64^ A nonfunctional glycine (G) was incorporated at the C-terminus during peptide synthesis to reduce the potential racemization of cysteine that is exacerbated by coupling cysteine directly to the resin. Cyclization was enforced by intramolecular oxidative disulfide bond formation between the sulfur atoms of the Cys residues that flank the aromatic/hydrophilic core sequence. As a result, the glycine residue is not incorporated in the cyclic peptide backbone but is an exocyclic appendage that serves no functional purpose for siRNA binding or delivery. CAPs were purified by HPLC and characterized by MALDI-TOF mass spectroscopy [see Materials and Methods and Supporting Information (Figures S1–S22 and Tables S1 and S2) for experimental details and characterization data].

### Correlation of siRNA Cellular Uptake Efficiency with CAP Sequence Identity

**Figure 2:**
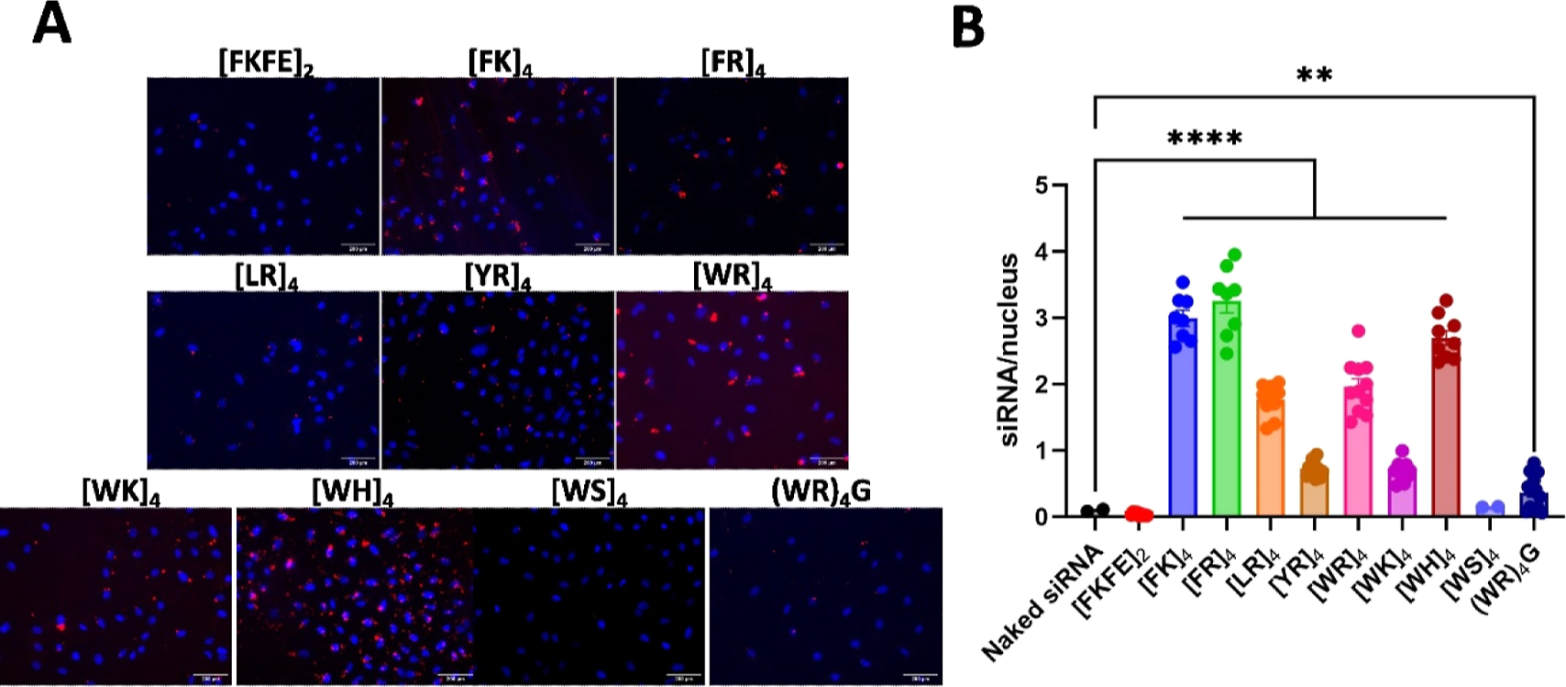
Cellular uptake of CAP–siRNA complexes into A549 lung adenocarcinoma cells. (A) Delivery of fluorescently labeled siRNA (siGlo Red-siRNA) with our CAPs in A549 lung cancer cells. Nuclei are stained with DAPI. Cell membranes are stained with Wheat Germ Agglutinin-Alexa 488. CAP concentration was 13.3 μM; siGlo Red-siRNA concentration was 132 nM. (B) Quantification of the number of CAP–siRNA complexes in A549 lung cancer cells. Amount of siRNA per nucleus represented as the average of at least two images (*n* = 2, **p ≤ 0.05, ****p ≤ 0.0001).

Next, we investigated the correlation between the CAP sequence and the efficiency of siRNA cytosolic translocation with each CAP–siRNA nanoparticle type. The impact of the CAP sequence on the efficiency of CAP–siRNA cellular uptake was determined by quantifying the amount of siRNA delivered to a cell using each CAP–siRNA complex, which was determined by analysis of fluorescence microscopy images of A549 cells incubated with fluorophore-labeled siRNA (siGlo Red-siRNA), a fluorescent plasma membrane stain (Wheat Germ Agglutinin), and a fluorescent nucleus stain (DAPI). Fluorescence microscope images were assessed, and the number of siRNA complexes per nucleus was determined by image processing in ImageJ/FIJI. The limit of this data is that the number of siRNA molecules in each instance of observable siGlo Red complex in each image cannot necessarily be attributed to a single siRNA. That is, these fluorescence features may be complexes that contain more than a single duplex of siRNA. Using this approach, we were able to assess the impact of the CAP amino acid sequence on the cellular uptake of siRNA as facilitated by CAP–siRNA complexes (Figures 2 and S45).

This data indicates that cationic charge improves uptake of CAP–siRNA complexes. Net neutral CAPs with N/P ratio <100, [WS]_4_ and [FKFE]_2_, have nearly negligible delivery to cells relative to any of the cationic CAPs. However, these results are unsurprising since it has been well-established in the general cell-penetrating peptide literature that cationic peptides, including oligo-arginine, TAT, and penetratin, are highly effective at facilitating cell membrane translocation.^67,68^

These data also suggest that disulfide-constrained cyclization improves CAP–siRNA uptake *in vitro*, which is demonstrated by improved cellular uptake of siRNA complexed with [WR]_4_ compared to (WR)_4_G, despite having nearly identical peptide sequences and N/P ratios (190). This data is in agreement with previous reports demonstrating increased cargo delivery with cyclization of other synthetic delivery vectors.^67,69,70^ Recent work by Liu et al. also shows a benefit to peptide disulfides for promoting interactions with type-II alveolar epithelial cells, suggesting specific membrane interactions.^71^ Additionally, these data validate the importance of charge over disulfide-constrained cyclization for promoting effective membrane translocation, indicated by improved siRNA translocation when complexed with (WR)_4_G than with both [FKFE]_2_ and [WS]_4_.

Although our data show that cationic charge is necessary for delivery, the CAP translocation of siRNA into the cytosol is also influenced by the identity of the aromatic residues of the CAP sequence. When the aromatic residue is Phe, Lys and Arg CAPs have similar siRNA translocation properties, which reflects the similar siRNA-binding affinities of [FK]_4_ and [FR]_4_. However, when the aromatic residue is Trp, siRNA translocation increases in the following order with regards to the cationic residue: Lys < Arg < His. When Arg is the cationic amino acid, the data show that siRNA delivery increases in the following order, with regards to the aromatic residue: Tyr < Trp < Phe. This trend deviates from the trend for CH–π hydrogen-bonding strength (His < Phe < Tyr < Trp).^72^ Interestingly, [WH]_4_, which utilizes histidine as both a partially cationic and aromatic side chain, translocated the most siRNA on average. This would suggest that although there is a slight decrease in positive charge, the ability of histidine to interact with cell–surface proteins may confer an additional advantage for these peptides to assist siRNA delivery. Furthermore, these trends cannot be attributed to N/P ratios because all CAPs included in this analysis have the same N/P ratio (∼190).

This analysis should be viewed with caution, however. For example, the high fluorescence intensity of the [WH]_4_ images may not accurately correlate with the actual number of counted siRNA complexes per nuclei. Since [WH]_4_ generates the largest complexes with siRNA (see Figures S41 and S44), it is likely that areas of high intensity represent clusters of several complexes that are counted as only a single data point. As a result, the level of details discussed in this analysis probably strains the strength of the data presented for siRNA translocation since images are not a strong method to quantify siRNA entry into cells. In the next section, we discuss quantitative functional knockdown of siRNA gene targets, which is a much more robust method to determine functional translocation efficiency.

### Effect of CAP Sequence on siRNA Binding

The impact of CAP sequence on the efficiency of CAP–siRNA complexation was determined by comparing the binding affinities of CAPs to a solution of FITC-labeled siRNA, containing 3–5 scrambled sequences, (FITC–siRNA) via a slot blot filtration assay (Figures S23–S33).^65^ Slot blot analysis enabled the extrapolation of the apparent dissociation constants, *K*_d_, for each sequence (Table 1). All CAPs demonstrated positive cooperative binding according to Hill slope values (*h* > 1), indicating that CAP sequence does not influence the type of binding interactions. We previously reported that complexation of siRNA with [WR]_4_ resulted in improved uptake *in vitro* and knockdown *in vivo* compared to siRNA–[FKFE]_2_ complexes.^55^ The apparent *K*_d_ values determined by slot blot analysis indicate that this likely is due to a 10-fold stronger siRNA-binding affinity for [WR]_4_ (2.8 μM) than for [FKFE]_2_ (29 μM). We hypothesized that the net neutral charge of [FKFE]_2_ reduces favorable interactions with the anionic siRNA payload. Therefore, we removed the glutamic acid residues of [FKFE]_2_ to generate the [FK]_4_ CAP with a net positive charge, which exhibited an increase in binding affinity to ∼11 μM. These results led us to probe the importance of the guanidinium ion of Arg compared to the ammonium ion of Lys in promoting favorable CAP–siRNA interactions using the [FR]_4_ CAP. A slight decrease in the apparent *K*_d_ was observed for [FR]_4_ (∼3 μM) compared to [FK]_4_. However, [WK]_4_ had a threefold increase in binding affinity (∼0.9 μM) compared to that of [WR]_4_ (∼3 μM). This suggests that exchanging K with R does not have a significant impact on apparent *K*_d_ when the hydrophobic residue is Phe but shows a slight preference for Lys when the hydrophobic residue is Trp. Generally, cationic character improved CAP–siRNA-binding affinity, as would be expected.

Next, we investigated the impact of the CAP hydrophobic and aromatic residues on siRNA binding. Aromatic amino acids have been shown to participate in CH–π hydrogen bonding with carbohydrates.^72−78^ We hypothesized that these types of interactions between aromatic CAP residues and siRNA ribose rings may play a role in CAP–siRNA complexation. Hudson *et al*. suggests that the strength of CH–π interactions increases in the following amino acid side chain order: H < F < Y < W.^72^ By comparing binding affinities of our hydrophobic/aromatic CAP variants, we observe a similar trend, with apparent *K*_d_ values decreasing in the order: [WH]_4_ > [FR]_4_ ∼ [FK]_4_ > [YR]_4_ > [WR]_4_ > [WK]_4_. Initially, we predicted that [WH]_4_ may have enhanced siRNA binding due to an increased probability of participating in CH–π interactions while maintaining a positive charge with the inclusion of His residues in the hydrophilic positions. Additionally, histidine residues have been shown to play important roles in a variety of oligonucleotide-binding interactions *in vivo*.^79−81^ However, our data indicate that [WH]_4_ binds more poorly than cationic Phe-, Tyr-, and Trp-containing sequences that include cationic Arg or Lys residues. This is consistent with ionic interactions in CAP–siRNA complexes being of primary importance for efficient binding since histidine is only partially positive at physiological pH, while both Lys and Arg residues are expected to be fully cationic (His p*K*_a_ = 6.04, Lys p*K*_a_ = 10.54, and Arg p*K*_a_ = 12.48).

The role of cationic charge and CH–π hydrogen bonding was further probed by testing [LR]_4_, which lacks the ability to participate in CH–π hydrogen bonding, and [WS]_4_, which is neutral but can still participate in CH–π hydrogen bonding. It should be noted that [LR]_4_ has a similar hydrophobicity to [FR]_4_ but lacks aromatic character. The binding affinity of [WS]_4_ (∼27 μM) is only slightly better than [FKFE]_2_, while [LR]_4_ (∼9 μM) revealed weakened binding compared to [WR]_4_ and [WK]_4_ CAPs but similar binding to [FK]_4_ and [FR]_4_ CAPs. These data further support the hypothesis that a cationic charge is essential to CAP–siRNA condensation. However, the impact of binding to siRNA with [LR]_4_ compared to aromatic CAPs suggests that putative CH–π interactions are nuanced, with Trp forming the most significant interactions with siRNA.

The importance of having a reversible, disulfide constraint has been previously validated using a variant of the [WR]_4_ peptide that was cyclized via a nonreducible thioether bond.^55^ Negligible gene knockdown in H441 cells with CAP–siRNA complexes in which the cyclization is not redox sensitive suggests that reductive cleavage and subsequent proteolytic degradation are necessary for efficient siRNA delivery.^55^ However, we wanted to further investigate the importance of peptide cyclization for siRNA binding. We prepared (WR)_4_G CAP (Ac-(WR)_4_G-NH_2_) that is a linear peptide to compare binding with the cyclic [WR]_4_ analogue. The (WR)_4_G peptide exhibits a binding affinity (30 μM) for siRNA lower than that of any of the CAPs assessed herein. The apparent *K*_d_ of the linear (WR)_4_G peptide is 10-fold lower than that of the cyclic [WR]_4_ (∼3 μM). Peptides patterned with alternating hydrophobic/hydrophilic residues are known to be prone to undergo self-assembly into peptide nanofibrils. Competitive self-assembly of the (WR)_4_G peptide may reduce the amount of peptide functionally available for siRNA complexation. In addition, the arrangement of the side chain residues in the cyclic form may also be more structurally accessible for cooperative siRNA interactions.

The nanoparticles that arise from CAP–siRNA condensation do not appear to have properties that strongly correlate with differences in the CAP sequence. As previously reported for [FKFE]_2_ and [WR]_4_ CAP–siRNA complexes,^55^ every CAP tested herein also formed nanoparticles that were ∼20 nm in diameter that aggregate into monodispersed clusters ∼200 nm in diameter as indicated by TEM images and DLS analysis (Table 1 and Figures S34–S44 in the Supporting Information). Nanoparticle morphologies were generally similar for all CAP–siRNA complexes, with no distinctive differences observed. Thus, the CAP sequence does not appear to strongly alter CAP–siRNA condensation structures at least at the limits of our ability to observe using the available data.

These binding analyses indicate several important features of CAPs that are essential for effective siRNA complexation to form CAP–siRNA nanoparticles. First, the cationic character is critically important for CAP–siRNA binding, presumably due to attractive charge interactions between cationic CAPs and negative phosphates in the siRNA cargo. A moderate enhancement of siRNA binding by CAP cations from Arg guanidinium cations is supported by some of the data, but ammonium cations from CAP Lys residues also enhance binding to siRNA. A strong preference between the two types of cations for CAP–siRNA nanoparticle formation cannot be strongly supported. While Arg-containing CAPs generally have stronger *K*_d_ values, the strongest siRNA binder in these studies is the [WK]_4_ CAP (∼0.9 μM) followed closely by the [WR]_4_ and [YR]_4_ CAPs (both ∼3 μM). Thus, the effect of cations is nuanced due to a significant effect of the aromatic groups in these peptides, with Trp-containing CAPs generally exhibiting stronger siRNA interactions than CAPs with Phe or nonaromatic residues. Thus, the strongly cationic character and the presence of aromatic residues that are competent to participate in CH–π interactions lead to CAP sequences that most effectively interact with siRNA.

### Functional Gene Knockdown by CAP–siRNA Complexes

**Figure 3:**
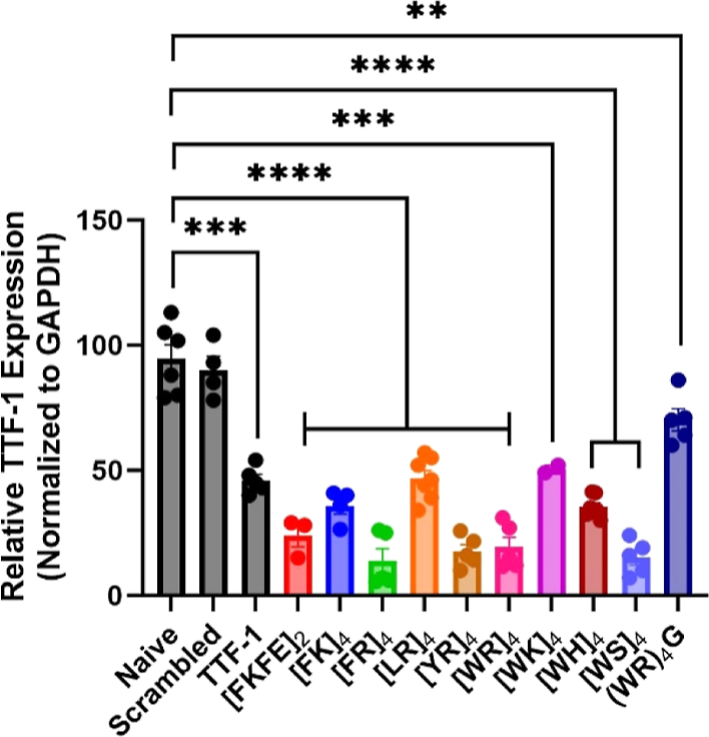
Knockdown efficiency of CAP–siRNA complexes against TTF-1 mRNA expression in A549 lung adenocarcinoma cells. Relative TTF-1 mRNA levels as percent knockdown normalized against GAPDH loading control determined by RT-qPCR. Scrambled is the control siRNA delivered with lipofectamine 3000. TTF-1 is delivery of TTF-1 siRNA with lipofectamine 3000 (*n* = 5, *p ≤ 0.05, **p ≤ 0.01, ***p ≤ 0.001).

Functional siRNA delivery must ultimately be determined by quantification of the knockdown efficacy of siRNA gene targets. To this end, we next assessed the functional gene knockdown of thymus transcription factor-1 (TTF-1) in A549 cells. This gene target and these cell types were chosen to maintain consistency with our initial report of siRNA delivery using CAP–siRNA complexation. TTF-1 mRNA expression was quantified against normalized GAPDH by utilizing complementary RT-qPCR (Figure 3).^82^ Additionally, we analyzed TTF-1 protein expression against total protein via Western blot analysis (Figure S46) and confirmed that knockdown experiments were conducted at nontoxic concentrations of peptide (Figure S47) to ensure that knockdown analyses were not due to cell death. Details of the experimental methods can be found in the Materials and Methods Section. Delivery of scrambled siRNA with lipofectamine resulted in no significant reduction in TTF-1 mRNA levels (∼10%), while delivery of TTF-1-specific siRNA with lipofectamine reduced TTF-1 mRNA levels by approximately 54%. These data demonstrate that [FKFE]_2_ and [WR]_4_ CAP–siRNA complexes both generate effective reduction of TTF-1 mRNA (∼80%), which is in agreement with previously reported data.^55^ The only CAPs tested that showed similar or slightly improved target knockdown compared to that of the original [FKFE]_2_ and [WR]_4_ peptides were [FR]_4_, [YR]_4_, and [WS]_4_. TTF-1 mRNA expression was reduced with [FK]_4_ and [WH]_4_ by approximately 70%, which is similar to the knockdown with lipofectamine. Additionally, increased CAP–siRNA binding affinity results in decreased knockdown efficiency. Improved TTF-1 silencing was observed with [FR]_4_ (∼86% knockdown, 12.07 μM *K*_d_) compared to [FK]_4_ (∼64% knockdown, ∼ 25.75 μM *K*_d_) and [WR]_4_ (∼80% knockdown, 4.85 μM *K*_d_) compared to [WK]_4_ (∼50% knockdown, 0.858 μM *K*_d_). These data suggest that the influence of cationic side chain identity on knockdown may also be dependent on the aromatic residue of the CAP sequence. Interestingly, reduction in mRNA (∼85% knockdown) by the [WS]_4_ peptide with a neutral charge and N/P ratio ∼0 was comparable to the [FR]_4_, [YR]_4_, and [WR]_4_ peptides. For cationic Trp-containing CAPs, TTF-1 silencing increases in the following order: [WK]_4_ < [WH]_4_ < [WR]_4_. Interestingly, the data also indicate a moderate reduction of mRNA levels with [LR]_4_ (∼50%) and (WR)_4_G (∼30%). TTF1 protein levels as measured by Western blot analysis were affected similarly by all CAP–siRNA complexes (∼50% decrease) without any significant differences, as were observed by measurement of mRNA levels (Figure S46). Protein knockdown measurements did not appear to be dependent upon siRNA delivery efficiency or mRNA knockdown. This discrepancy between mRNA levels and protein levels is likely due to protein turnover and other regulatory mechanisms of TTF-1 expression. CAPs with high levels of mRNA knockdown could potentially be used at lower dosage while maintaining functional protein knockdown.^83−85^

This knockdown analysis indicates several important trends for effective functional siRNA delivery *in vitro*. First, we demonstrate that the disulfide constraint in our CAP system is essential for improving functional knockdown. As expected, (WR)_4_G facilitates less-effective gene silencing than its cyclic peptide counterparts, further demonstrating that the disulfide constraint of CAPs offers an advantage over linear sequences. Overall, we can conclude that CAPs containing arginine as the cationic residue demonstrate improved TTF-1 knockdown compared to their lysine-containing counterparts and lipofectamine. Additionally, most cyclic CAPs containing both arginine and aromatic residues facilitate >80% TTF-1 silencing. The exception to this observation is [WK]_4_, which demonstrated more moderate mRNA knockdown (∼50%, still as effective as the lipofectamine control). We hypothesize that the nanomolar binding affinity of [WK]_4_ with siRNA prohibits the effective release of the siRNA upon cellular delivery, reducing the amount of siRNA available to the RNAi machinery. Interestingly, [WS]_4_ was one of the most effective CAPs for TTF-1 mRNA silencing (∼85% knockdown). Similarly, [FKFE]_2_ also demonstrated excellent mRNA knockdown efficiency (∼80%), even though the CAPs are net neutral, demonstrated weak siRNA binding (≥30 μM), and exhibited lower levels of delivery of siRNA *in vitro*. The performance of these CAPs supports the hypothesis that weaker binding to siRNA allows for a more efficient release upon cellular internalization. As was observed with siRNA internalization experiments, there appears to be no clear correlation between the knockdown efficiency and N/P ratio of these CAP–siRNA complexes. Generally, although with some exceptions as noted, strongly cationic character and the presence of aromatic residues that are competent to participate in CH–π interactions lead to CAP–siRNA complexes that most effectively promote gene silencing.

### Evaluation of Uptake Mechanism of CAP–siRNA Complexes

**Figure 4:**
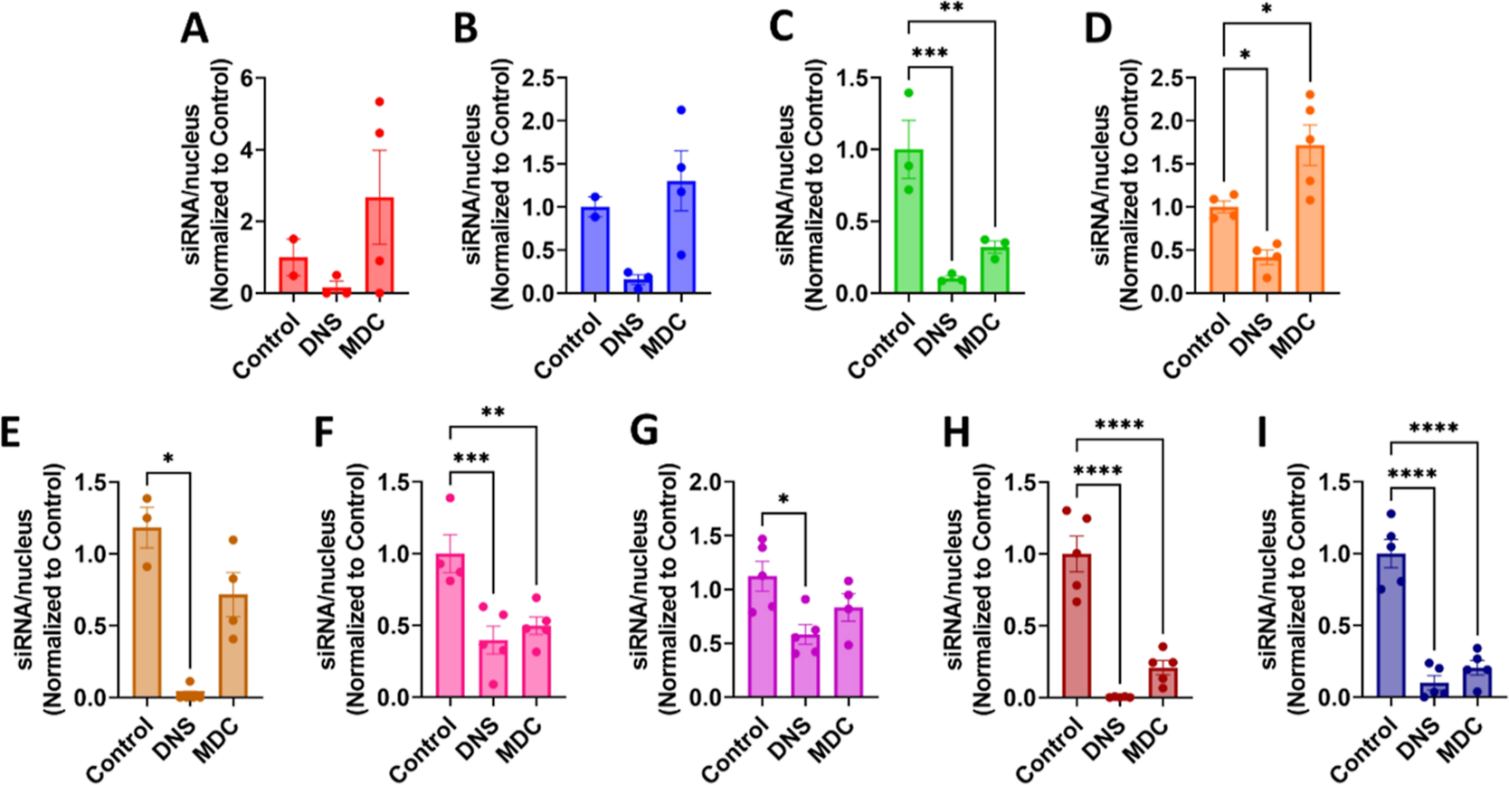
Quantification of siRNA/nucleus in the presence of DNS (400 μM) or MDC (200 μM) in A549 lung adenocarcinoma cells. (A) [FKFE]_2_ (*n* = 2), (B) [FK]_4_ (*n* = 2), (C) [FR]_4_ (*n* = 3, **p ≤ 0.01, ***p ≤ 0.001), (D) [LR]_4_ (*n* = 4, *p ≤ 0.05), and (E) [YR]_4_ (*n* = 3, *p ≤ 0.05). (F) [WR]_4_ (*n* = 4, **p ≤ 0.01, ***p ≤ 0.001), (G) [WK]_4_ (*n* = 5, *p ≤ 0.05), (H) [WH]_4_ (*n* = 5, ****p ≤ 0.0001), and (I) (WR)_4_G (*n* = 5, ****p ≤ 0.0001).

**Figure 5:**
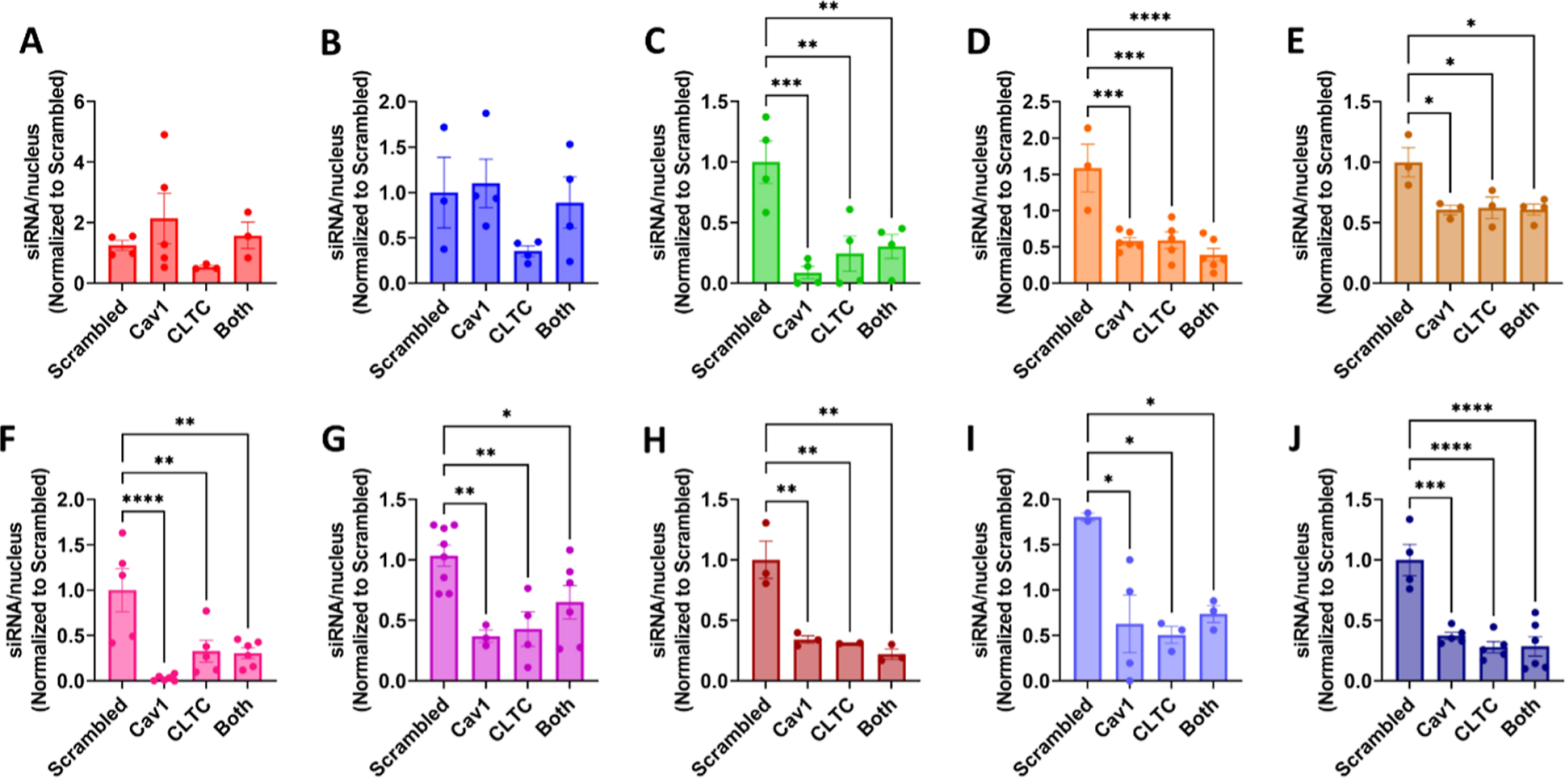
Quantification of siRNA/nucleus in A549 lung adenocarcinoma cells previously treated with scrambled, Cav1, CLTC, or Cav1 + CLTC siRNA. (A) [FKFE]_2_ (*n* = 4), (B) [FK]_4_ (*n* = 3), (C) [FR]_4_ (*n* = 4, **p ≤ 0.01, ***p ≤ 0.001), (D) [LR]_4_ (*n* = 3, ***p ≤ 0.001, ****p ≤ 0.0001), (E) [YR]_4_ (*n* = 3, *p ≤ 0.05), (F) [WR]_4_ (*n* = 5, **p ≤ 0.01, ***p ≤ 0.001), (G) [WK]_4_ (*n* = 7, *p ≤ 0.05, **p ≤ 0.01), (H) [WH]_4_ (*n* = 3, **p ≤ 0.01), (I) [WS]_4_ (*n* = 2, *p ≤ 0.05), and (J) (WR)_4_G (*n* = 4, ***p ≤ 0.001, ****p ≤ 0.0001).

We next sought insight into the mechanism of siRNA uptake facilitated by CAP–siRNA nanoparticles. The main pathways of endocytosis in lung epithelia are caveolin- and clathrin-mediated endocytosis,^59^ which both rely on dynamin for vesicle internalization. Pathway characterization can be determined by pharmaceutical inhibition^59,86−89^ or genetic knockdown of respective pathways, with inhibition of CAP–siRNA complexes indicating which pathway is likely utilized. Our first approach was to utilize known inhibitors of different endocytic pathways to determine if our complexes utilize clathrin (CDE) or caveolin-mediated pathways (CME) (Figures 4 and S48–S49). The drugs chosen for these studies were DNS, which acts to inhibit dynamin proteins involved in endocytic vesicle pinching^88,90^ and monodansylcadaverine (MDC).^91,92^ At low concentrations, DNS primarily inhibits CDE; however, at high concentrations, the drug also disrupts lipid raft-dependent uptake necessary for CME. MDC is another inhibitor of CDE, which stabilizes nascent clathrin-coated vesicles, limiting formation of new clathrin lattices.^93^ Additionally, MDC may act to suppress activator Rho GTPases important in actin-dependent macropinocytosis.^94^ We further confirmed uptake pathway by electroporation delivery of siRNA to knock down caveolin-1 (Cav-1), the primary component of caveolae,^95^ and/or clathrin heavy chain (CLTC), an essential protein for the formation of clathrin-coated vesicles and pits (Figures 5 and S50 and S51).^96,97^

Inhibition and endocytic pathway knockdown were shown by reduced A594-holotransferrin uptake (hTf) and reduced BODIPY-lactosyl ceramide (LacCer) uptake for clathrin-mediated and caveolae-mediated inhibition, respectively, with knockdown confirmed by Western blot analysis (Figure S52). The CAP–siRNA complexes were delivered to cells ± inhibitors or pathway knockdown. Results of the delivery of CAP–siRNA nanoparticles to A549 cells pre-exposed to low concentrations of DNS, high concentrations of DNS, or MDC are outlined in Figure 4. All CAP–siRNA complexes demonstrated reduced uptake under DNS inhibition, suggesting that a dynamin-mediated mechanism is responsible for CAP–siRNA complex uptake; however, MDC produced varying results. Transfection with [FKFE]_2_, [FK]_4_, [YR]_4_, and [WK]_4_ resulted in no change in siRNA uptake, indicating that stabilization of nascent clathrin-coated vesicles may not be essential and that actin-dependent micropinocytosis is not a utilized pathway. Decreased siRNA uptake with [FR]_4_, [LR]_4_, [WR]_4_, [WH]_4_, and (WR)_4_G in cells exposed to MDC suggests that a clathrin-mediated or micropinocytosis pathway is utilized. Although there is variability in the effect of MDC on siRNA transfection, taken together, these results suggest that our CAPs utilize clathrin- or caveolin-mediated endocytosis.

Knockdown of Cav-1, CLTC, or both reduced uptake of all CAP–siRNA complexes except for [FKFE]_2_ and [FK]_4_ (Figure 5). Knockdown of CLTC reduced the level of internalization of [FKFE]_2_ and [FK]_4_ complexes, while knockdown of Cav1 or Cav1 + CLTC resulted in no change in complex internalization. Therefore, we can conclude that [FKFE]_2_ and [FK]_4_ nanoparticles are predominantly utilizing CDE, while the remaining CAPs are utilizing a combination of CDE and CME. Additionally, the size range of CAP–siRNA complexes at the ratio used for delivery experiments was 135–291 nm (Figures S34–S44 and Table 1). Therefore, we can conclude that the complex size does not correlate with a particular pathway or delivery efficiency. Further experimentation and understanding of the interactions between these complexes and the cell membrane would be necessary to elucidate why a given CAP–siRNA nanoparticle uses given pathway(s). Although we have demonstrated these trends in A549 cells, endocytic pathways may occur via different mechanisms in other cell types. Therefore, analysis of other endocytic pathways, such as macropinocytosis,^59^ Cdc42, CLIC/GEEC pathways, and membrane ruffling creating transient nanopores^60,61^ may be necessary when expanding these CAPs to different applications.

These data invite further investigation into the mode of endosomal escape of the CAP–siRNA complexes. Recently, Pei and coworkers demonstrated that a cyclic CPP containing Phe, Trp, and Arg residues facilitated endosomal escape by a vesicle budding and collapse mechanism.^98^ The authors tested the method of endosomal escape of a known CPP (CPP12) by conjugation to a pH-sensitive fluorescent dye (pHAb) that demonstrates strong fluorescence in the acidic environment of the endosomes and lysosomes but is only weakly fluorescent in the cytosol.^98^ Using confocal microscopy, the authors were able to demonstrate that the CPP formed clusters along the endosomal membrane, which resulted in budding events that detach from the intact endosome and ultimately collapse in the cytosol.^98^ Additionally, the authors concluded that endosomal escape efficiency of a given CPP is dependent on the frequency of CPP-induced budding and collapse events.^98^ The CAPs discussed herein may induce endosomal budding and collapse events in a similar manner, although a detailed examination of these mechanisms is beyond the scope of this report.

## Conclusions

Here, we have demonstrated that a complex relationship exists between CAP–siRNA binding kinetics, translocation efficiency of siRNA, gene target silencing, and the endocytic uptake mechanism. Strong cationic character and the presence of aromatic residues that are competent to participate in CH–π interactions lead to CAP sequences that most effectively interact with siRNA. By comparing binding affinities of our hydrophobic/aromatic CAP variants, we observed apparent *K*_d_ values decreasing in the following order: [WH]_4_ > [FR]_4_ ∼ [FK]_4_ > [YR]_4_ > [WR]_4_ > [WK]_4_. Additionally, the weak binding of net neutral [WS]_4_ suggests that the cationic character is essential for effective CAP–siRNA condensation. While Arg-containing CAPs generally have improved binding affinity, the strongest binder in these studies is [WK]_4_. We also demonstrate that cationic charge improves the uptake of CAP–siRNA complexes *in vitro*. Net neutral CAPs [WS]_4_ and [FKFE]_2_ had negligible uptake relative to any cationic CAP. Disulfide-constrained cyclization improved uptakes, which is demonstrated by improved internalization of [WR]_4_–siRNA complexes compared to that of [WR]_4_G–siRNA complexes. Additionally, aromatic residue identity influences the efficiency of CAP–siRNA translocation across the cell membrane, particularly for tryptophan-containing sequences.

Functional delivery of siRNA and knockdown, efficiency is generally improved when utilizing arginine-containing sequences with aromatic amino acids, including Phe, Trp, and Tyr, further implicating the importance of CH–π and charge interactions for CAP–siRNA condensation and membrane permeation^48,99^ and endosomal escape of the CAP–siRNA particles,^100,101^ which was demonstrated with [FR]_4_, [YR]_4_, and [WR]_4_. However, the knockdown efficiency of these complexes is more strongly correlated with CAP–siRNA binding affinity. Although [WK]_4_ demonstrated significant delivery of siRNA, poor knockdown (∼50%) is potentially a result of strong complex binding that prevents release of siRNA for processing by the RNAi machinery. Additionally, [FKFE]_2_, [FR]_4_, and [WS]_4_ demonstrated effective knockdown (>80%) even though these CAPs had minimal siRNA delivery. The significantly lower binding affinity of these CAPs with the siRNA likely allows for all siRNA delivered into the cytosol to be available for processing by the RNAi machinery. Last, we demonstrated that most CAPs participate in a combination of clathrin- and caveolin-dependent pathways in A549 cells. Our data suggests that [LR]_4_ exclusively participates in caveolin-mediated uptake while [FKFE]_2_, [FR]_4_, and [WS]_4_ exclusively participate in clathrin-mediated uptake. For most of our CAPs, the ability to participate in multiple uptake pathways improves targeted gene silencing, but the high knockdown efficiency of [FKFE]_2_ and [WS]_4_ emphasizes the importance of clathrin-mediated endocytosis.

These characteristics represent key design principles for the refinement of cell-penetrating peptide systems for siRNA delivery. The work described herein also provides critical insight into the mechanistic pathways by which CAPs facilitate endocytic delivery of functional siRNA. Although we have demonstrated that CAPs analyzed in this study primarily utilize clathrin- and caveolin-mediated uptake, their utilization of these pathways may be cell-specific, warranting further investigation of other endocytic pathways, such as macropinocytosis,^59^ Cdc42, CLIC/GEEC pathways, and membrane ruffling creating transient nanopores^60,61^ in other cell lines. The variation in the endocytic pathway favored by different CAP–siRNA complexes suggests that there may be tissue-/cell-type tropisms *in vivo* that would require further investigation. Due to variations in CAP–siRNA complex size (∼130–250 nm, Table 2), the route of administration may also influence effective functional delivery *in vivo* and in clinical settings.^62^ Therefore, the addition of cell-/tissue-specific targeting ligands may improve localized delivery and minimization of off-target effects *in vivo* and be required for effective clinical outcomes; however, addition of these ligands may have secondary effects on siRNA-binding and endocytic uptake mechanism.^102,103^ CAPs remain a promising class of cell-penetrating peptides for functional delivery of siRNA cargo, and the insights presented herein reveal key design principles for the optimization of these materials that will facilitate translational applications for *in vivo* gene knockdown and ultimately clinical applications.

**Table 2:** Dynamic Light Scattering^a^

CAP	CAP–siRNA particle cluster radius (nm)	PDI	count rate (*kCnt*/s)
[FKFE]_2_	188 ± 11	0.38 ± 0.10	609.5 ± 65.2
[FK]_4_	247 ± 35	0.40 ± 0.10	837.5 ± 96.0
[FR]_4_	212 ± 25	0.31 ± 0.08	716.9 ± 81.3
[LR]_4_	209 ± 25	0.30 ± 0.08	541.4 ± 47.1
[YR]_4_	135 ± 18	0.30 ± 0.10	620.8 ± 31.5
[WR]_4_	202 ± 20	0.41 ± 0.08	2180.5 ± 333.1
[WK]_4_	135 ± 20	0.28 ± 0.05	595.5 ± 44.6
[WH]_4_	210 ± 30	0.41 ± 0.07	1933.3 ± 368.6
[WS]_4_	291 ± 33	0.28 ± 0.06	548.0 ± 44.0
(WR)_4_G	189 ± 9	0.32 ± 0.07	2054.2 ± 570.9

a Radii, polydispersity index (PDI), and count rate (*kCnt*/s) of CAP–siRNA nanoparticles were measured via DLS and are reported as the average of at least three measurements with the error reported as the standard error of the mean.

## Supporting Information Available

Peptide purification and characterization data, nitrocellulose and nylon images for binding assays, TEM images of peptides and peptide–siRNA complexes, and supporting data for siRNA cell internalization mechanism experiments (PDF)
