# Supplementary material for: Impact of Peptide Sequence on Functional siRNA Delivery and Gene Knockdown with Cyclic Amphipathic Peptide Delivery Agents
Source: Mol Pharm. 2023 Nov 14;20(12):6090–103. doi: 10.1021/acs.molpharmaceut.3c00455 (PMC10698724; doi:10.1021/acs.molpharmaceut.3c00455)
Supplement: Supplementary file 1 — mp3c00455_si_001.pdf [file mp3c00455_si_001.pdf]

Impact of Peptide Sequence on Functional siRNA Delivery and Gene Knockdown with Cyclic Amphipathic Peptide Delivery Agents

Melissa L. Jagrosse,[a] Uday K. Baliga,[c] Christopher W. Jones,[a] Jade J. Russell,[a] Claudia I. García,[a] Rauf Ahmad Najar,[c] Arshad Rahman,[c] David A. Dean,[c] and Bradley, L. Nilsson[a,b]*

[a]Department of Chemistry, University of Rochester, NY 14627-0216, USA.

[b]Materials Science Program, University of Rochester, Rochester, NY 14627, USA.

[c]Department of Pediatrics and Neonatology, University of Rochester Medical Center, School of Medicine and Dentistry, University of Rochester, Rochester, NY 14642, USA.

E-mail: bradley.nilsson@rochester.edu

Tel. +1 585 276-3053

Supporting Information

Table of Contents

Figure S1. Chemical structures of CAPs. **(A)** [FKFE]$_2$, **(B)** [FK]$_4$, **(C)** [FR]$_4$, **(D)** [LR]$_4$, **(E)** [YR]$_4$, **(F)** [WR]$_4$, **(G)** [WK]$_4$, **(H)** [WH]$_4$, **(I)** [WS]$_4$, and **(J)** (WR)$_4$G. 5
Table S1. Analytical HPLC retention times (R$_t$, determined by the apex of the relevant peaks in the HPLC analyses shown in Figure S2) and mobile phase conditions for purified CAPs............6
Figure S2. Analytical HPLC traces of purified CAPs observed and using mobile and stationary phase conditions described in **Table S1**. **(A)** [FKFE]$_2$, **(B)** [FK]$_4$, **(C)** [FR]$_4$, **(D)** [LR]$_4$, **(E)** [YR]$_4$, **(F)** [WR]$_4$, **(G)** [WK]$_4$, **(H)** [WH]$_4$, **(I)** [WS]$_4$, and **(J)** (WR)$_4$G. 7
Figure S3. Concentration curve for purified [FKFE]$_2$. 8
Figure S4. Concentration curve for purified [FK]$_4$. 8
Figure S5. Concentration curve for purified [FR]$_4$. 9
Figure S6. Concentration curve for purified [LR]$_4$. 9
Figure S7. Concentration curve for purified [YR]$_4$. 10
Figure S8. Concentration curve for purified [WR]$_4$. 10
Figure S9. Concentration curve for purified [WK]$_4$. 11
Figure S10. Concentration curve for purified [WH]$_4$. 11
Figure S11. Concentration curve for purified [WS]$_4$. 12
Figure S12. Concentration curve for purified (WR)$_4$G. 12

Table S2. Calculated and observed m/z data from MALDI-TOF-MS for purified CAPs...........13
Figure S13. MALDI-TOS-MS spectra for purified $[FKFE]_2$. 13
Figure S14. MALDI-TOS-MS spectra for purified $[FK]_4$...14
Figure S15. MALDI-TOS-MS spectra for purified $[FR]_4$...14
Figure S16. MALDI-TOS-MS spectra for purified $[LR]_4$...15
Figure S17. MALDI-TOS-MS spectra for purified $[YR]_4$. 15
Figure S18. MALDI-TOS-MS spectra for purified $[WR]_4$. 16
Figure S19. MALDI-TOS-MS spectra for purified $[WK]_4$. 16
Figure S20. MALDI-TOS-MS spectra for purified $[WH]_4$. 17
Figure S21. MALDI-TOS-MS spectra for purified $[WS]_4$...17
Figure S22. MALDI-TOS-MS spectra for purified $(WR)_4G$...18
Figure S23. Nitrocellulose (left) and nylon (right) membranes for $[FKFE]_2$. 19
Figure S24. Nitrocellulose (left) and nylon (right) membranes for $[FK]_4$..................................19
Figure S25. Nitrocellulose (left) and nylon (right) membranes for $[FR]_4$..................................19
Figure S26. Nitrocellulose (left) and nylon (right) membranes for $[LR]_4$..................................20
Figure S27. Nitrocellulose (left) and nylon (right) membranes for $[YR]_4$. 20
Figure S28. Nitrocellulose (left) and nylon (right) membranes for $[WR]_4$. 20
Figure S29. Nitrocellulose (left) and nylon (right) membranes for $[WK]_4$. 21
Figure S30. Nitrocellulose (left) and nylon (right) membranes for $[WH]_4$. 21
Figure S31. Nitrocellulose (left) and nylon (right) membranes for $[WS]_4$. 21
Figure S32. Nitrocellulose (left) and nylon (right) membranes for $(WR)_4G$. 22
Figure S33. Plots of fraction bound (siRNA) vs. [CAP], (μM) obtained from slot blot filtration assay. **(A)** $[FKFE]_2$, **(B)** $[FK]_4$, **(C)** $[FR]_4$, **(D)** $[LR]_4$, **(E)** $[YR]_4$, **(F)** $[WR]_4$, **(G)** $[WK]_4$, **(H)** $[WH]_4$, **(I)** $[WS]_4$, and **(J)** $(WR)_4G$. 23
Figure S34. Nanoparticles characteristics of $[FKFE]_2$ complexed with siRNA. Transmission electron micrographs of **(A)** 1000-times peptide:siRNA excess and **(B)** 10-times peptide:siRNA excess. Dynamic light scattering plot in **(C)** 1000-times peptide:siRNA excess and **(D)** 10-times peptide:siRNA excess..24
Figure S35. Nanoparticles characteristics of $[FK]_4$ complexed with siRNA. Transmission electron micrographs of **(A)** 1000-times peptide:siRNA excess and **(B)** 10-times peptide:siRNA excess. Dynamic light scattering plot in **(C)** 1000-times peptide:siRNA excess and **(D)** 10-times peptide:siRNA excess..24
Figure S36. Nanoparticles characteristics of $[FR]_4$ complexed with siRNA. Transmission electron micrographs of **(A)** 1000-times peptide:siRNA excess and **(B)** 10-times peptide:siRNA excess. Dynamic light scattering plot in **(C)** 1000-times peptide:siRNA excess and **(D)** 10-times peptide:siRNA excess..25
Figure S37. Nanoparticles characteristics of $[LR]_4$ complexed with siRNA. Transmission electron micrographs of **(A)** 1000-times peptide:siRNA excess and **(B)** 10-times peptide:siRNA excess. Dynamic light scattering plot in **(C)** 1000-times peptide:siRNA excess and **(D)** 10-times peptide:siRNA excess..25
Figure S38. Nanoparticles characteristics of $[YR]_4$ complexed with siRNA. Transmission electron micrographs of **(A)** 1000-times peptide:siRNA excess and **(B)** 10-times peptide:siRNA excess. Dynamic light scattering plot in **(C)** 1000-times peptide:siRNA excess and **(D)** 10-times peptide:siRNA excess..26
Figure S39. Nanoparticles characteristics of $[WR]_4$ complexed with siRNA. Transmission electron micrographs of **(A)** 1000-times peptide:siRNA excess and **(B)** 10-times peptide:siRNA

excess. Dynamic light scattering plot in **(C)** 1000-times peptide:siRNA excess and **(D)** 10-times peptide:siRNA excess. 26

Figure S40. Nanoparticles characteristics of $[WK]_4$ complexed with siRNA. Transmission electron micrographs of **(A)** 1000-times peptide:siRNA excess and **(B)** 10-times peptide:siRNA excess. Dynamic light scattering plot in **(C)** 1000-times peptide:siRNA excess and **(D)** 10-times peptide:siRNA excess. 27

Figure S41. Nanoparticles characteristics of $[WH]_4$ complexed with siRNA. Transmission electron micrographs of **(A)** 1000-times peptide:siRNA excess and **(B)** 10-times peptide:siRNA excess. Dynamic light scattering plot in **(C)** 1000-times peptide:siRNA excess and **(D)** 10-times peptide:siRNA excess. 27

Figure S42. Nanoparticles characteristics of $[WS]_4$ complexed with siRNA. Transmission electron micrographs of **(A)** 1000-times peptide:siRNA excess and **(B)** 10-times peptide:siRNA excess. Dynamic light scattering plot in **(C)** 1000-times peptide:siRNA excess and **(D)** 10-times peptide:siRNA excess. 28

Figure S43. Nanoparticles characteristics of $(WR)_4G$ complexed with siRNA. Transmission electron micrographs of **(A)** 1000-times peptide:siRNA excess and **(B)** 10-times peptide:siRNA excess. Dynamic light scattering plot in **(C)** 1000-times peptide:siRNA excess and **(D)** 10-times peptide:siRNA excess. 28

Figure S44. Radii of CAP-siRNA complexes at 1000-times peptide:siRNA excess. Statistical significance was evaluated using one-way ANOVA ($n = 9$, $^*p \leq 0.05$). 29

Figure S45. Delivery of fluorescently labelled siRNA (red) with CAPs in A549 lung adenocarcinoma cells. Nuclei are stained with DAPI (blue). Cell membranes are stained with Wheat Germ Agglutinin-Alexa 488 (green). CAP concentrations were 13.3 µM; siGlo Red-siRNA concentrations were 132 nM. 29

Figure S46. Knockdown efficiency of CAP-siRNA complexes against TTF-1 protein expression in A549 lung adenocarcinoma cells by Western Blot densitometry analysis**.** **(A)** Representative Western Blot TTF-1 protein expression against total protein in A549 lung adenocarcinoma cells exposed to no siRNA (naïve), lipofectamine-siRNA complexes, or CAP-siRNA complexes. **(B)** Relative TTF-1 protein expression as percent knockdown normalized to total protein determined by Western Blot densitometry analysis. Scrambled is control siRNA delivered with lipofectamine. TTF-1 is delivery of TTF-1 siRNA with lipofectamine ($n = 2$, $^*p \leq 0.05$). 30

Figure S47. Cytotoxicity analysis of lipofectamine and CAPs (13.3 µM). **(A)** 4 h and **(B)** 24 h after dosing ($n = 4$, $^{****}p \leq 0.0001$). 30

Figure S48. Images used to assess siRNA delivery efficiency of CAP-siRNA complexes in A549 lung adenocarcinoma cells in the presence of DNS (400 µM) or MDC (200 µM). siRNA is labelled with Cy3 (red). Nuclei are stained with DAPI (blue). Cell membranes are stained with Wheat Germ Agglutinin-Alexa 488 (green). 31

Figure S49. Images used to assess siRNA delivery efficiency of CAP-siRNA complexes in A549 lung adenocarcinoma cells in the presence of DNS (400 µM) or MDC (200 µM). siRNA is labelled with Cy3 (red). Nuclei are stained with DAPI (blue). 32

Figure S50. Delivery of CAP-siRNA complexes in $Cav1^+/CLTC^+$ (Scrambled), $Cav1^-/CLTC^+$ (Cav1), $Cav1^+/CLTC^-$ (CLTC), or $Cav1^-/CLTC^-$ (Cav1+CLTC) A549 adenocarcinoma cells. siRNA is labelled with Cy3 (red). Nuclei are stained with DAPI (blue). Cell membranes are stained with Wheat Germ Agglutinin-Alexa 488 (green). 33

Figure S51. Delivery of CAP-siRNA complexes in Cav1^{+}/CLTC^{+} (Scrambled), Cav1^{-}/CLTC^{+} (Cav1), Cav1^{+}/CLTC^{-} (CLTC), or Cav1^{-}/CLTC^{-} (Cav1+CLTC) A549 adenocarcinoma cells. siRNA is labelled with Cy3 (red). Nuclei are stained with DAPI (blue). 33
Figure S52. Western blot densitometry analysis verifying Cav1, CLTC, or Cav1+CLTC knockdown in A549 lung adenocarcinoma cells. **(A)** Relative Cav1 expression as percent knockdown normalized to GAPDH (n = 3, $**p \leq 0.01$). **(B)** Relative CLTC expression as percent knockdown normalized to GAPDH (n = 3, $*p \leq 0.05$)...34

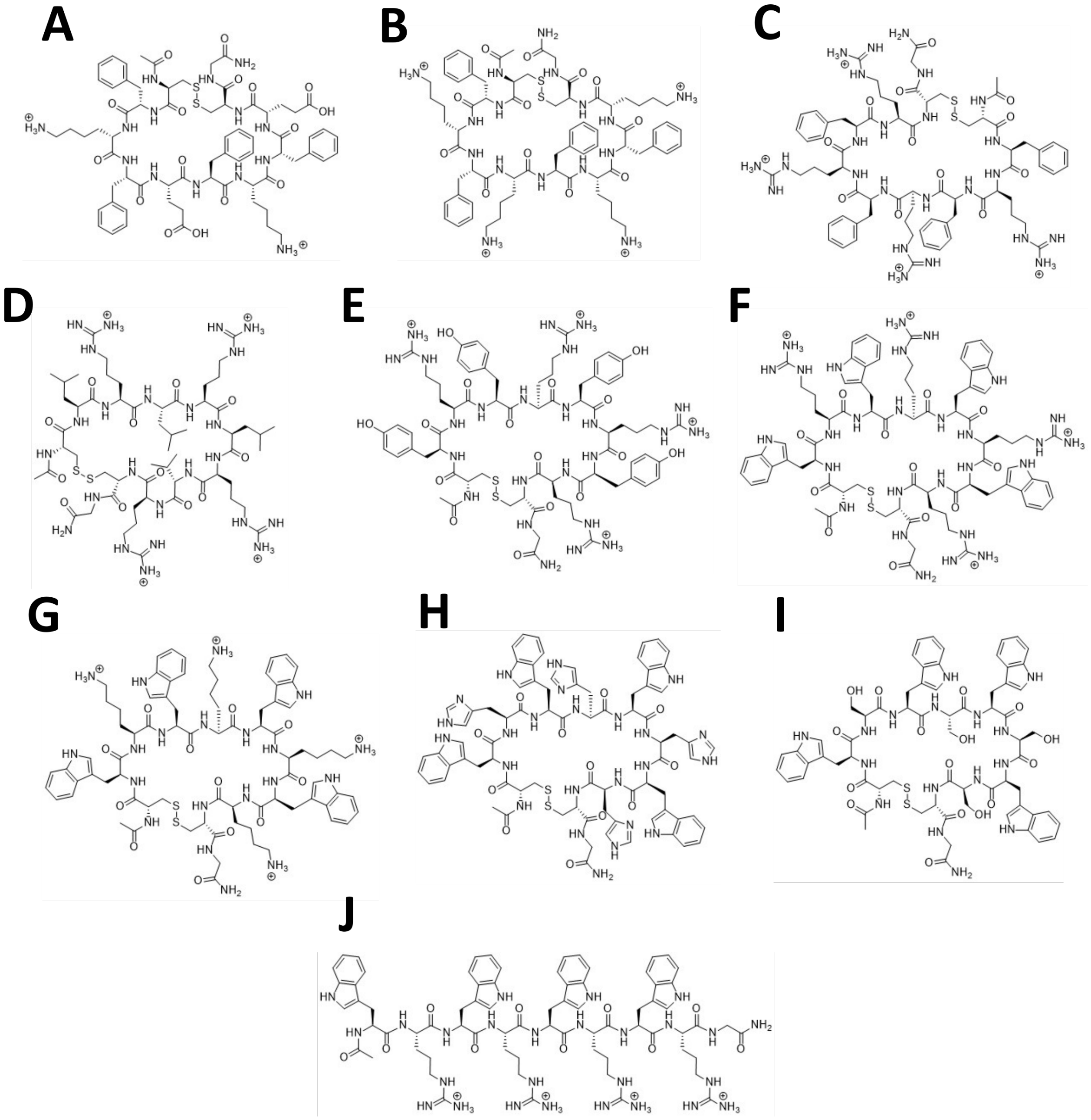

Figure S1. Chemical structures of CAPs. **(A)** [FKFE]$_2$, **(B)** [FK]$_4$, **(C)** [FR]$_4$, **(D)** [LR]$_4$, **(E)** [YR]$_4$, **(F)** [WR]$_4$, **(G)** [WK]$_4$, **(H)** [WH]$_4$, **(I)** [WS]$_4$, and **(J)** (WR)$_4$G.

Table S1. Analytical HPLC retention times (R_t, determined by the apex of the relevant peaks in the HPLC analyses shown in Figure S2) and mobile phase conditions for purified CAPs.

**Peptide**	**R_t** (min)	**Gradient (solution** A**: water/0.5% TFA; solution** B**: acetonitrile/0.5% TFA)**
L-Ac-C[FKFE]$_2$CG-NH$_2$	11.86	Isocratic 5% **B** 5 min, 5-95% **B** over 10min, 95% **B** 5 min, flow rate = 100 mL/min
L-Ac-C[FK]$_4$CG-NH$_2$	11.35	Isocratic 5% **B** 5 min, 5-95% **B** over 10min, 95% **B** 5 min, flow rate = 100 mL/min
L-Ac-C[FR]$_4$CG-NH$_2$	11.29	Isocratic 5% **B** 5 min, 5-95% **B** over 10min, 95% **B** 5 min, flow rate = 100 mL/min
L-Ac-C[LR]$_4$CG-NH$_2$	11.21	Isocratic 5% **B** 5 min, 5-95% **B** over 10min, 95% **B** 5 min, flow rate = 100 mL/min
L-Ac-C[YR]$_4$CG-NH$_2$	10.47	Isocratic 5% **B** 5 min, 5-95% **B** over 10min, 95% **B** 5 min, flow rate = 100 mL/min
L-Ac-C[WR]$_4$CG-NH$_2$	11.62	Isocratic 5% **B** 5 min, 5-95% **B** over 10min, 95% **B** 5 min, flow rate = 100 mL/min
L-Ac-C[WK]$_4$CG-NH$_2$	11.24	Isocratic 5% **B** 5 min, 5-95% **B** over 10min, 95% **B** 5 min, flow rate = 100 mL/min
L-Ac-C[WS]$_4$CG-NH$_2$	14.07	Isocratic 5% **B** 5 min, 5-95% **B** over 10min, 95% **B** 5 min, flow rate = 100 mL/min
L-Ac-C[WH]$_4$CG-NH$_2$	11.43	Isocratic 5% **B** 5 min, 5-95% **B** over 10min, 95% **B** 5 min, flow rate = 100 mL/min
L-Ac-(WR)$_4$G-NH$_2$	11.59	Isocratic 5% **B** 5 min, 5-95% **B** over 10min, 95% **B** 5 min, flow rate = 100 mL/min

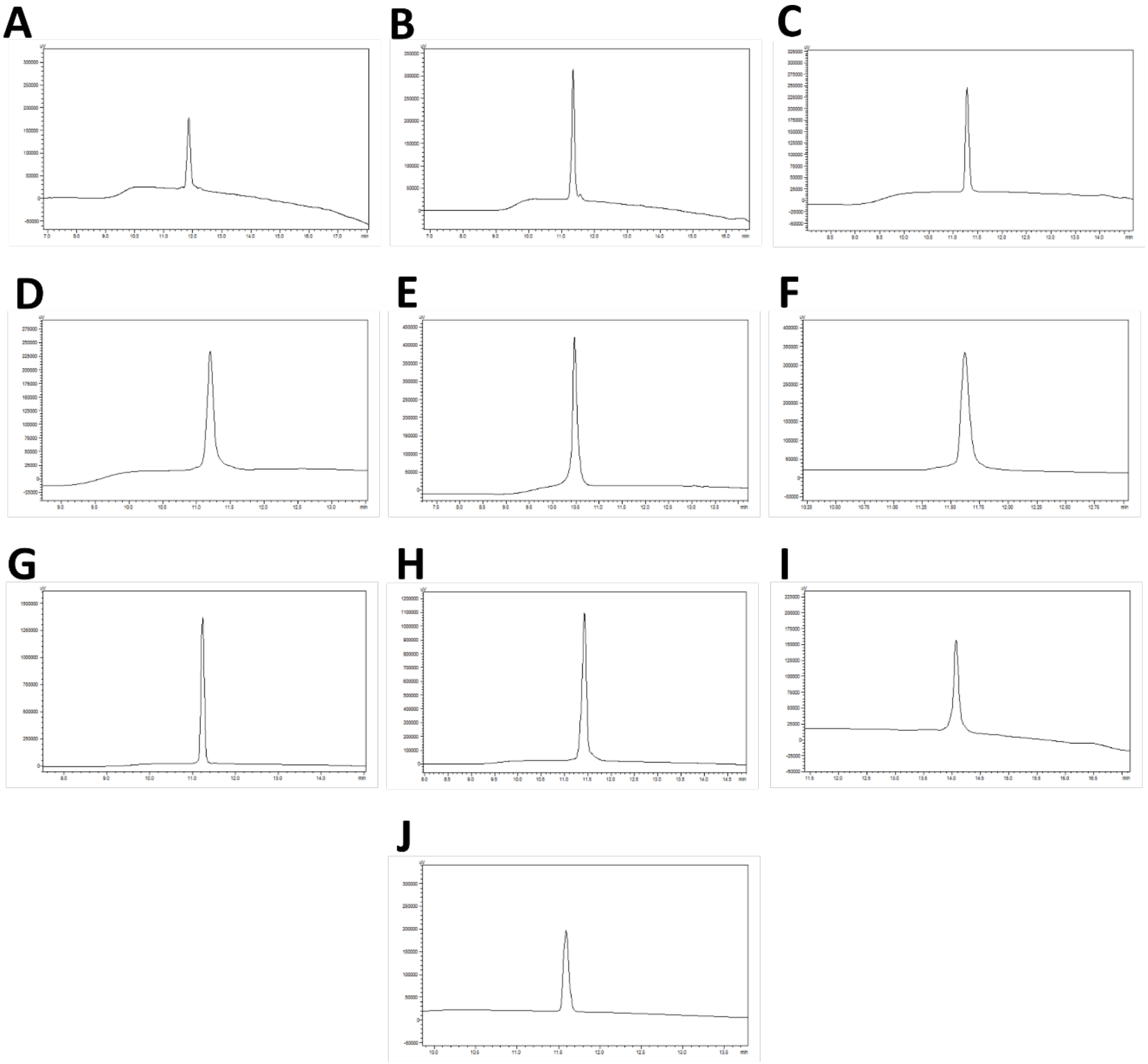

Figure S2. Analytical HPLC traces of purified CAPs observed and using mobile and stationary phase conditions described in **Table S1**. **(A)** $[FKFE]_2$, **(B)** $[FK]_4$, **(C)** $[FR]_4$, **(D)** $[LR]_4$, **(E)** $[YR]_4$, **(F)** $[WR]_4$, **(G)** $[WK]_4$, **(H)** $[WH]_4$, **(I)** $[WS]_4$, and **(J)** $(WR)_4G$.

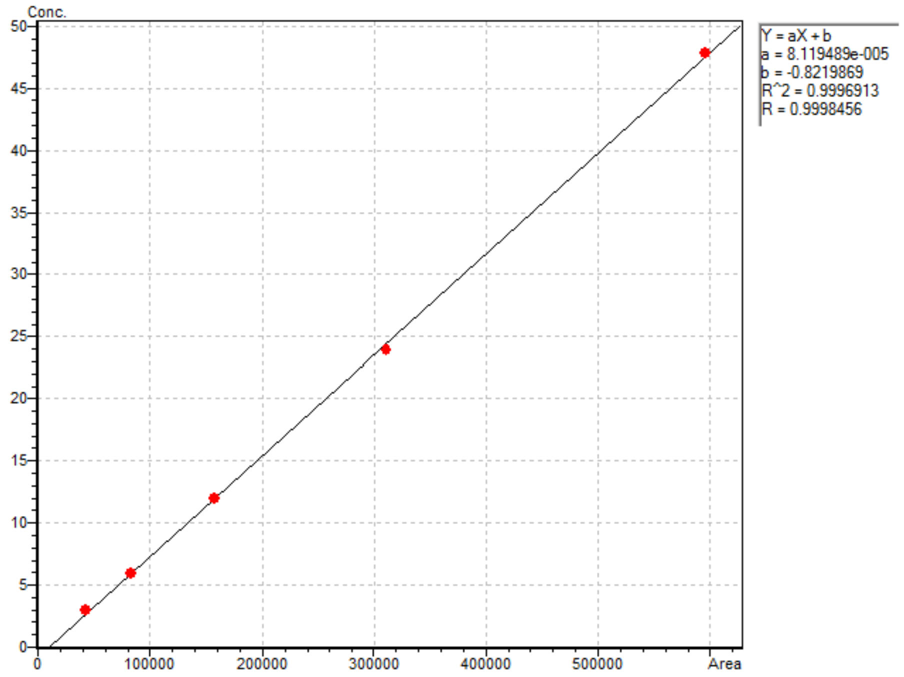

Figure S3. Concentration curve for purified $[FKFE]_2$.

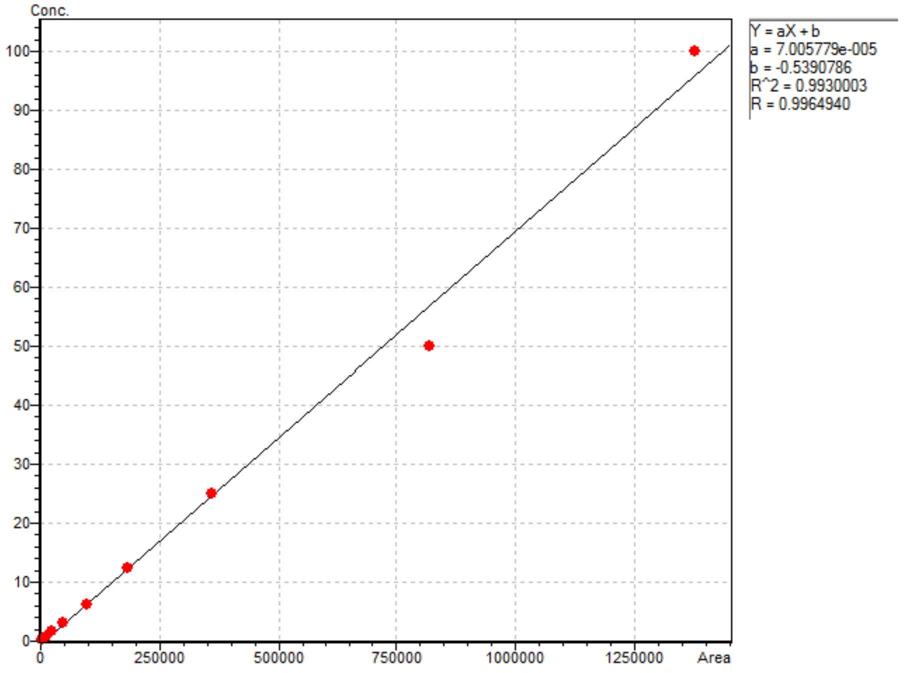

Figure S4. Concentration curve for purified $[FK]_4$.

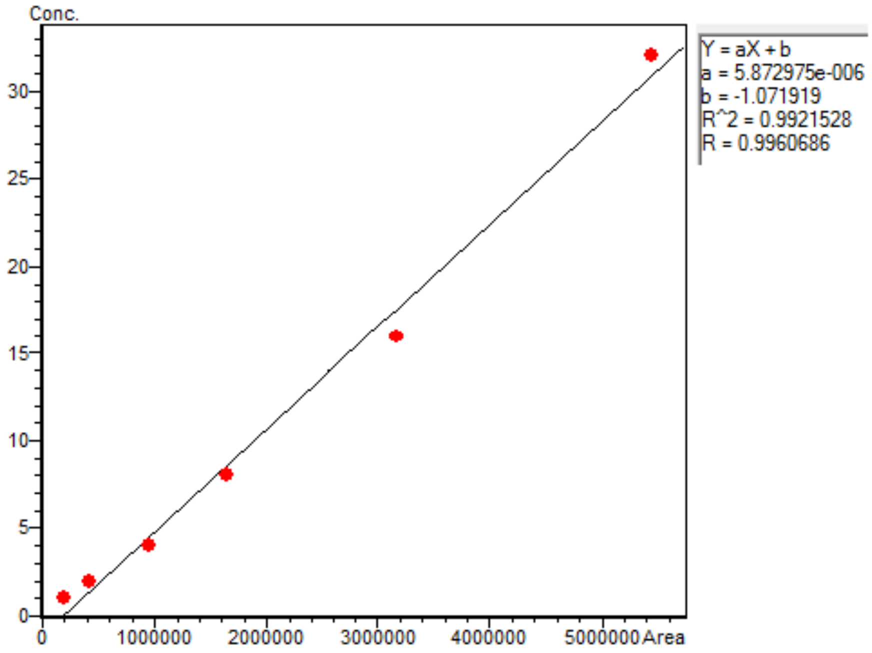

Figure S5. Concentration curve for purified [FR]$_4$.

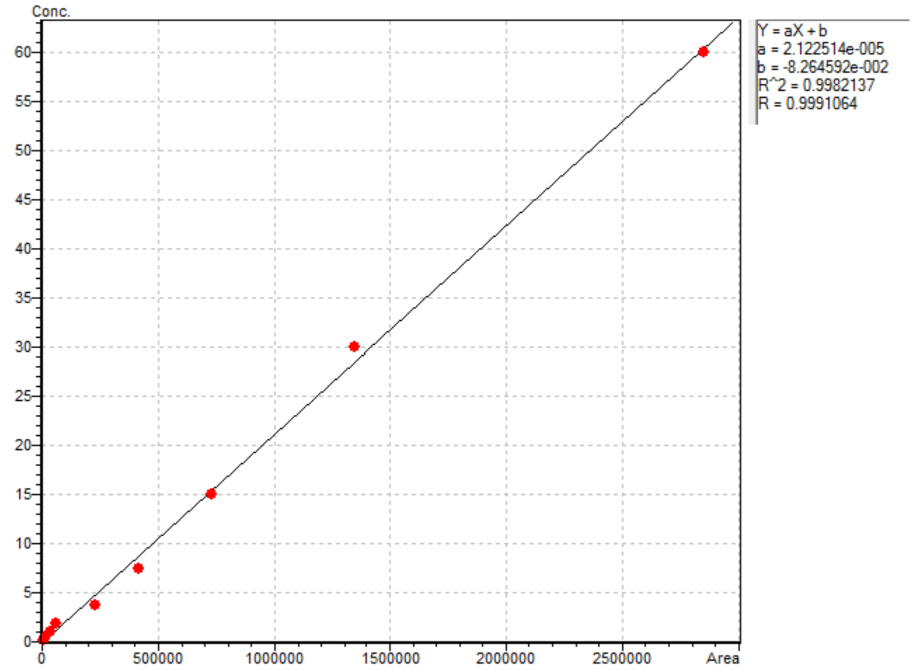

Figure S6. Concentration curve for purified [LR]$_4$.

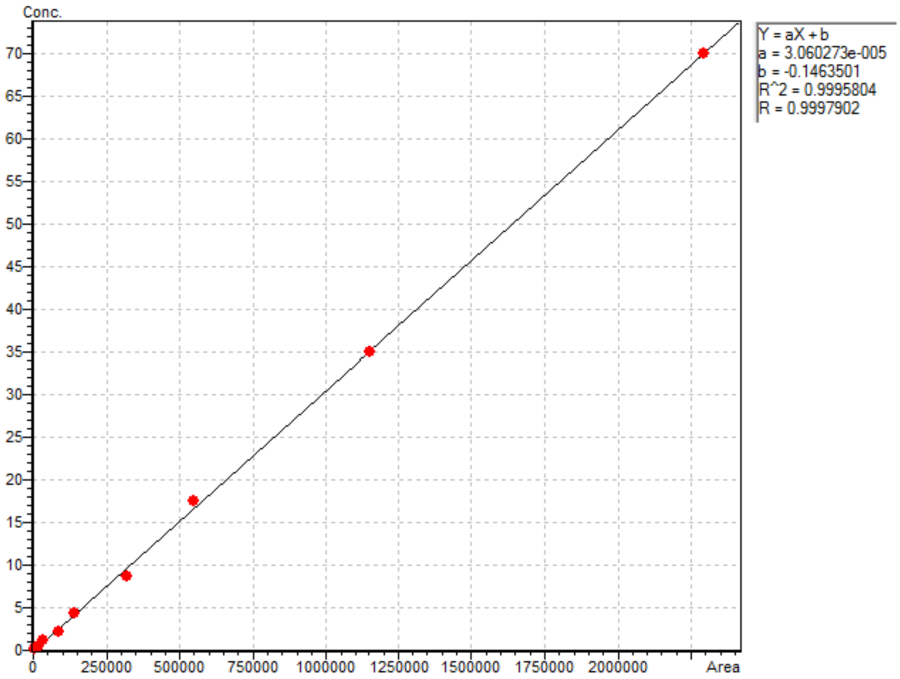

Figure S7. Concentration curve for purified $[YR]_4$.

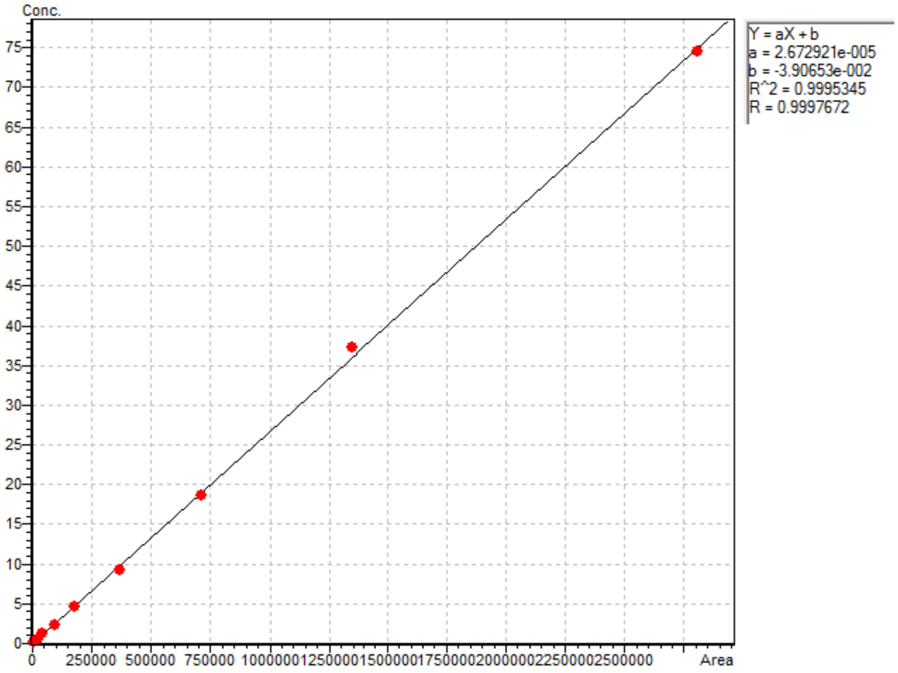

Figure S8. Concentration curve for purified $[WR]_4$.

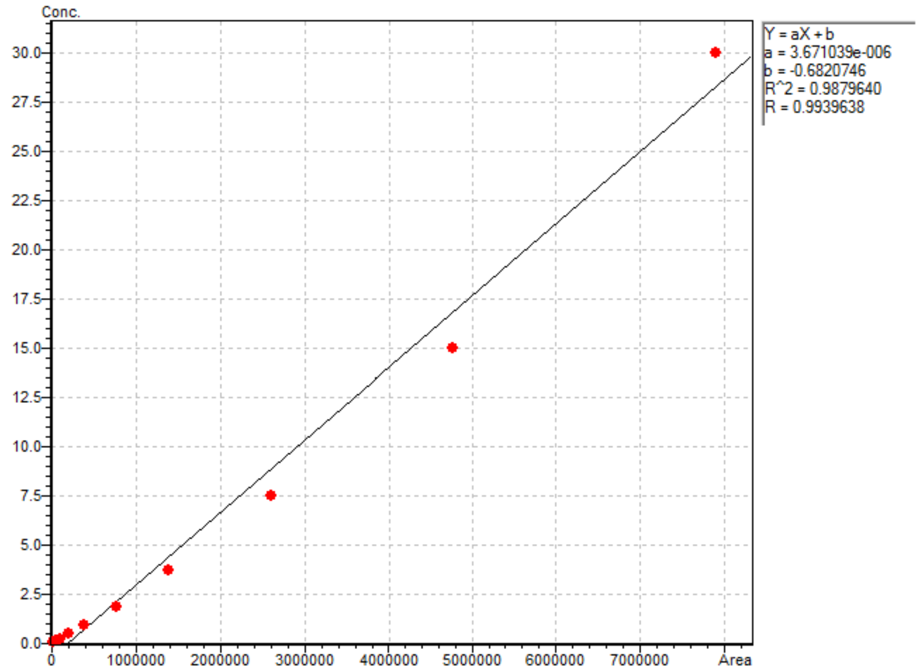

Figure S9. Concentration curve for purified $[WK]_4$.

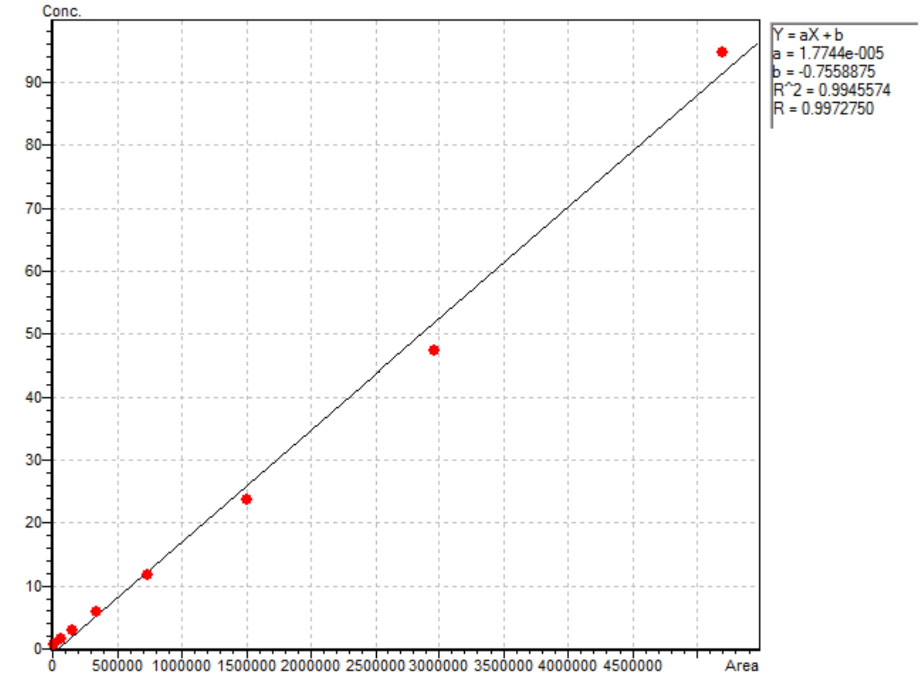

Figure S10. Concentration curve for purified $[WH]_4$.

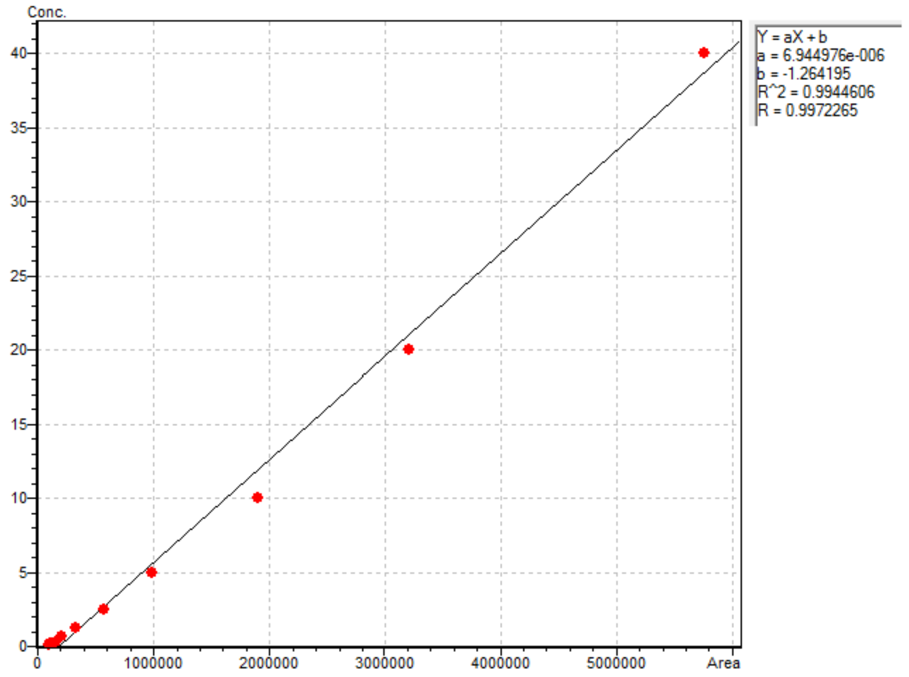

Figure S11. Concentration curve for purified $[WS]_4$.

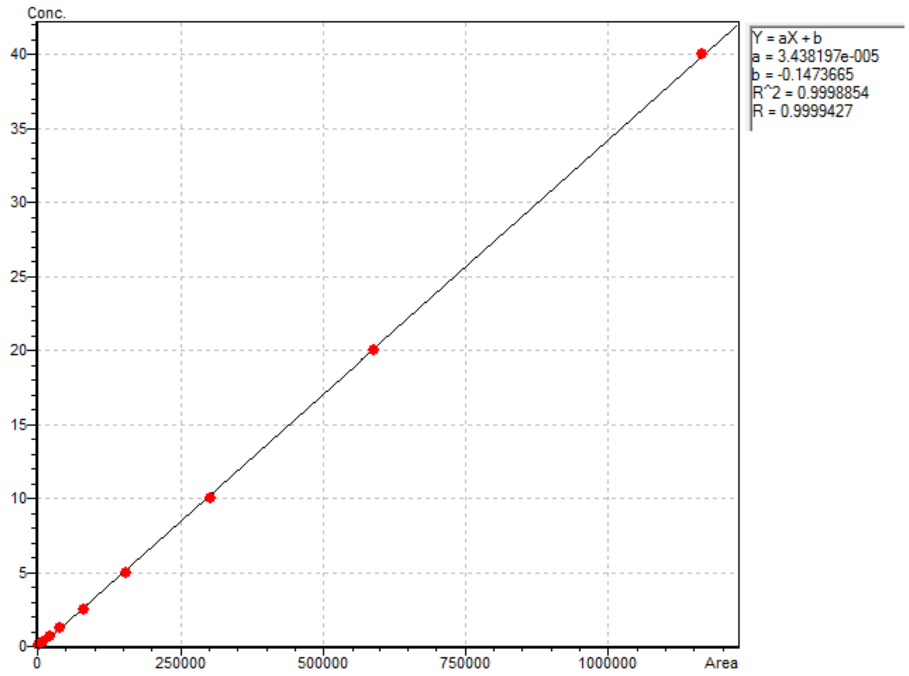

Figure S12. Concentration curve for purified $(WR)_4G$.

Table S2. Calculated and observed m/z data from MALDI-TOF-MS for purified CAPs.

Peptide	Calc. [MH+]	Calc. [MNa+]	Calc. [MK+]	Obs. [MH+]	Obs. [MNa+]	Obs. [MK+]
Ac-C[FKFE]$_2$CG	1424.62	1447.61	1463.72	1423.9501	1445.8917	1471.8314
Ac-C[FK]$_4$CG	1424.74	1447.73	1463.84	1423.3742	-	-
Ac-C[FR]$_4$CG	1536.77	1559.76	1575.87	1535.2476	1559.2835	-
Ac-C[LR]$_4$CG	1396.80	1419.79	1435.9	1398.5431	1420.6031	-
Ac-C[YR]$_4$CG	1600.75	1623.74	1639.85	1600.7138	1620.7284	-
Ac-C[WR]$_4$CG	1692.81	1715.80	1731.91	1692.8695	-	-
Ac-C[WK]$_4$CG	1580.79	1603.78	1619.89	1579.6159	1601.6778	1618.6555
Ac-C[WS]$_4$CG	1412.51	1435.5	1451.61	-	1438.0690	1453.1329
Ac-C[WH]$_4$CG	1615.80	1638.79	1654.9	1614.2797	1637.3608	1654.2778
Ac-(WR)$_4$G	1488.81	1511.8	1527.91	1487.8181	-	-

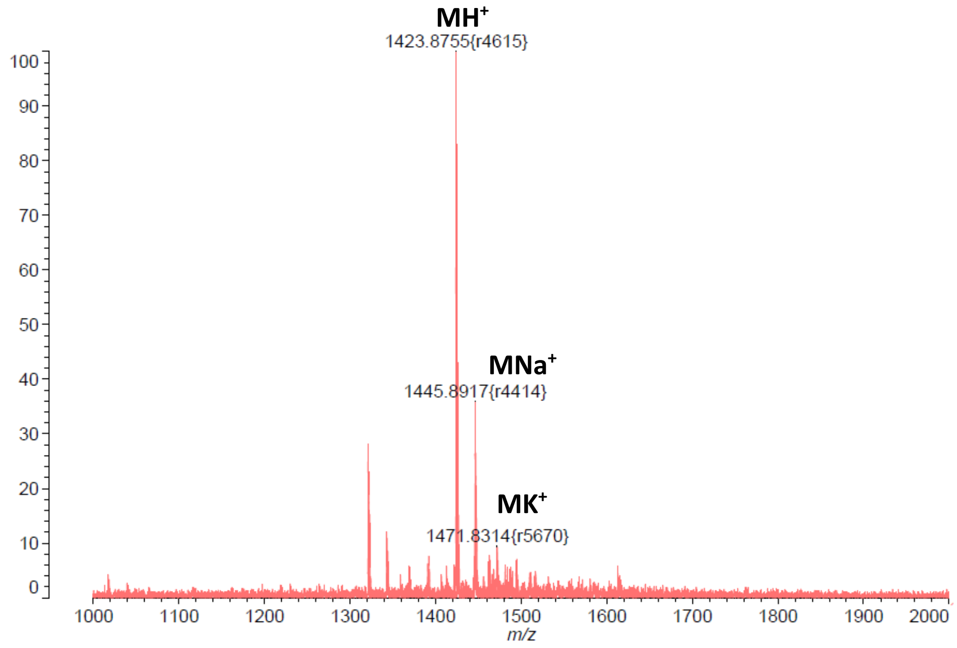

Figure S13. MALDI-TOS-MS spectra for purified [FKFE]$_2$.

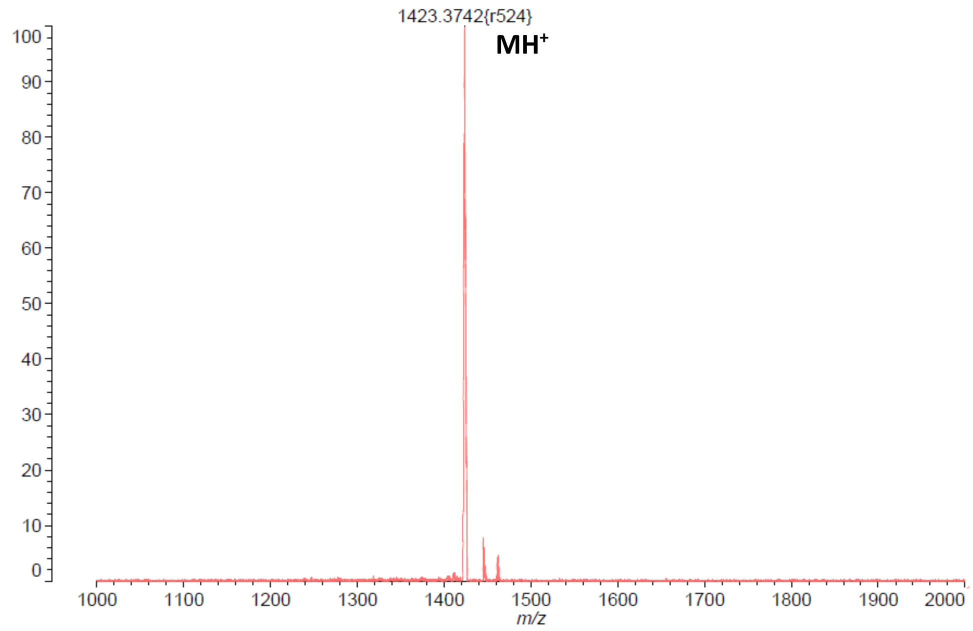

Figure S14. MALDI-TOS-MS spectra for purified $[FK]_4$.

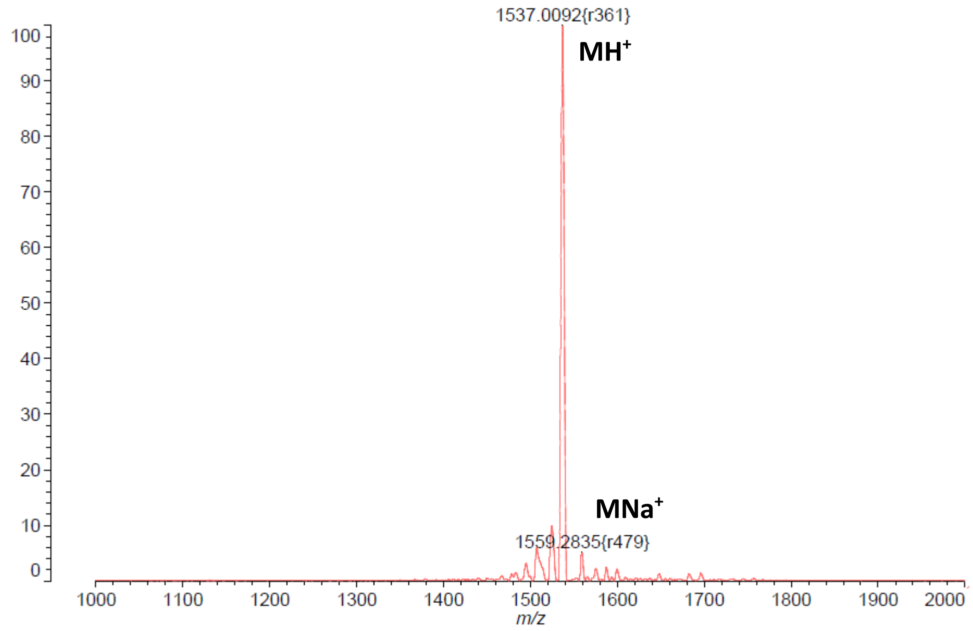

Figure S15. MALDI-TOS-MS spectra for purified $[FR]_4$.

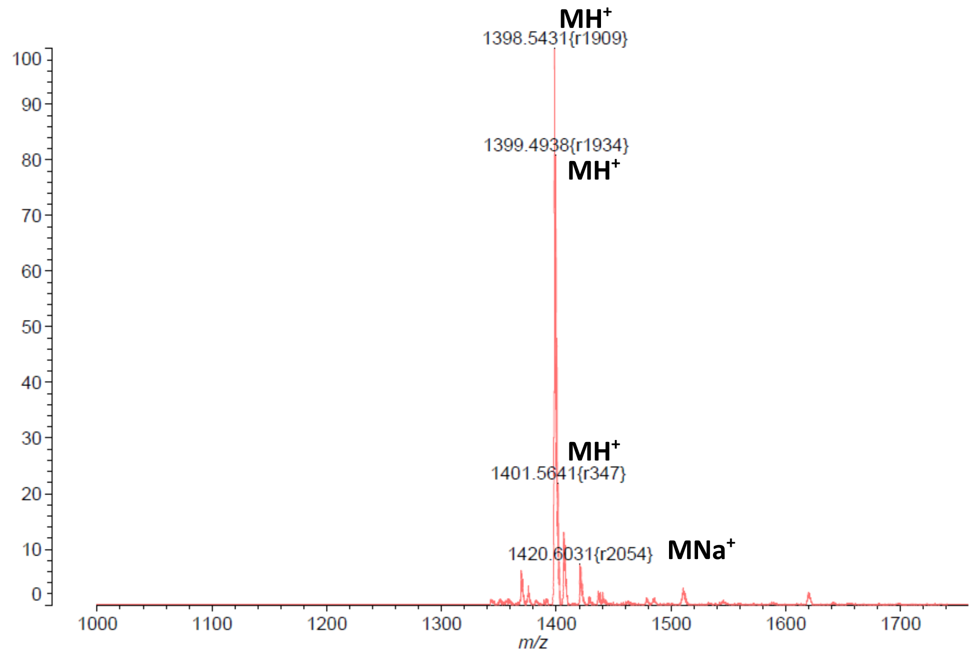

Figure S16. MALDI-TOS-MS spectra for purified [LR]4.

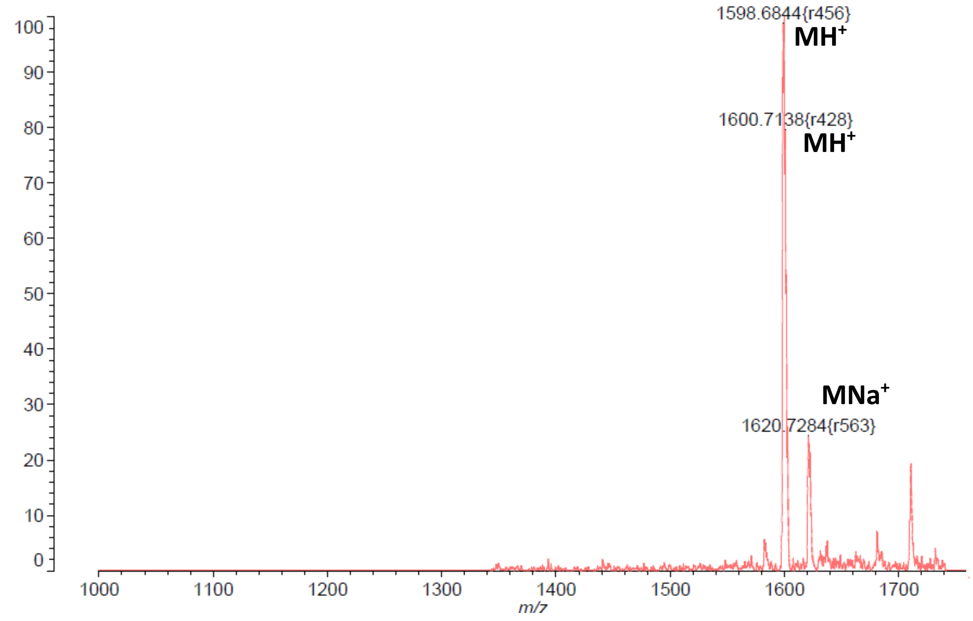

Figure S17. MALDI-TOS-MS spectra for purified [YR]4.

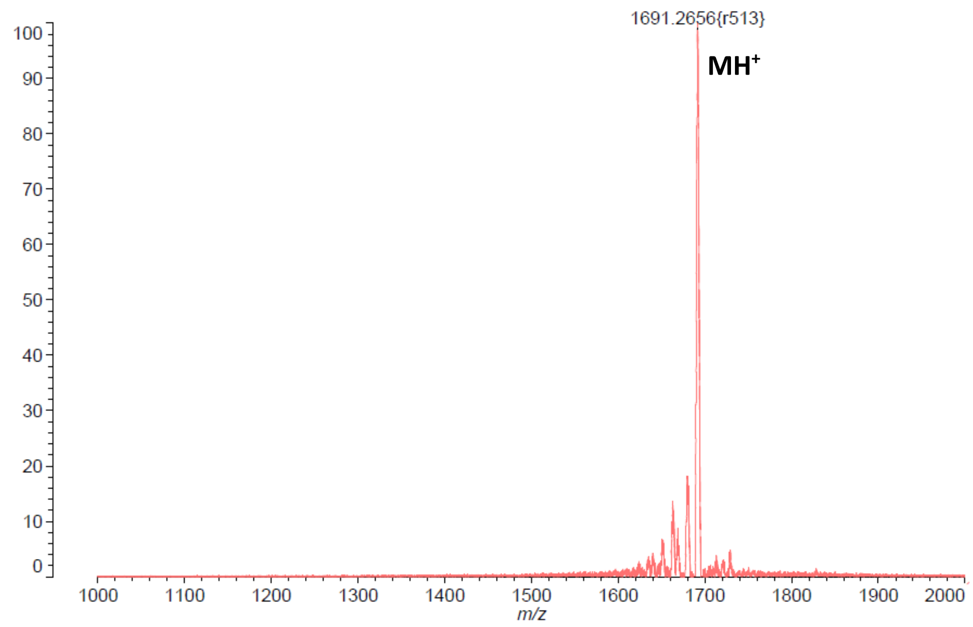

Figure S18. MALDI-TOS-MS spectra for purified $[WR]_4$.

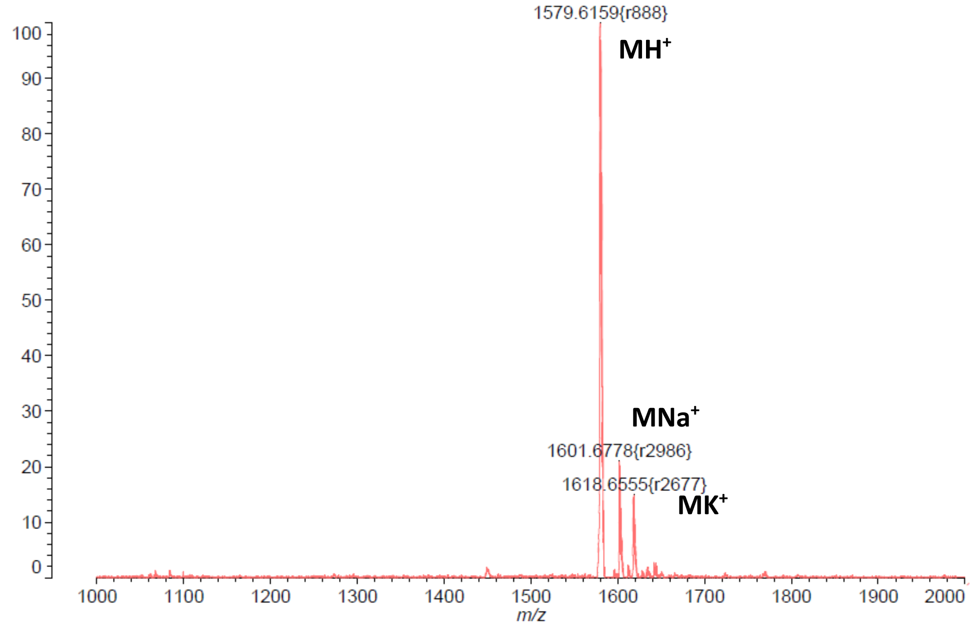

Figure S19. MALDI-TOS-MS spectra for purified $[WK]_4$.

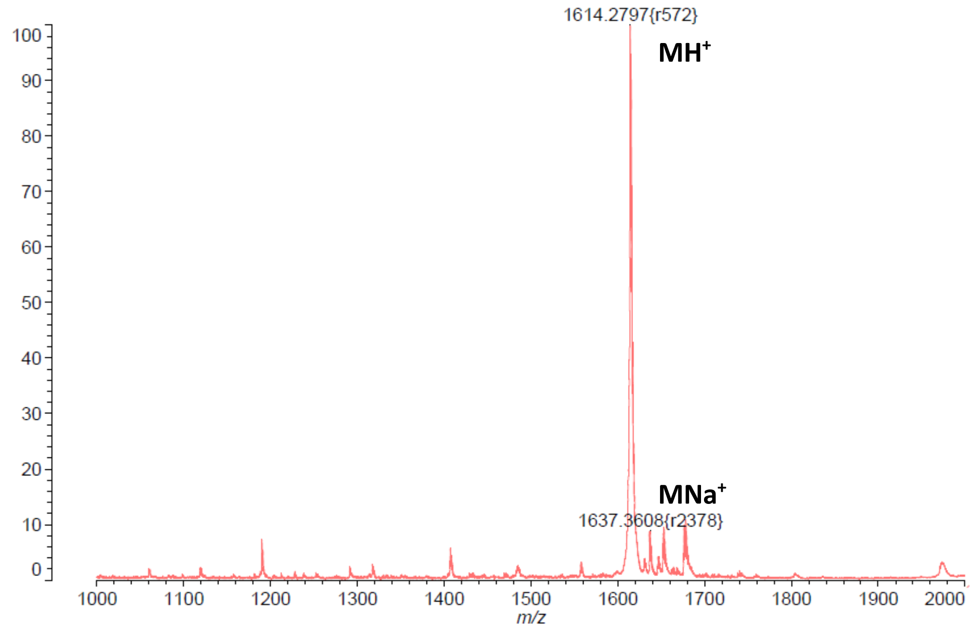

Figure S20. MALDI-TOS-MS spectra for purified $[WH]_4$.

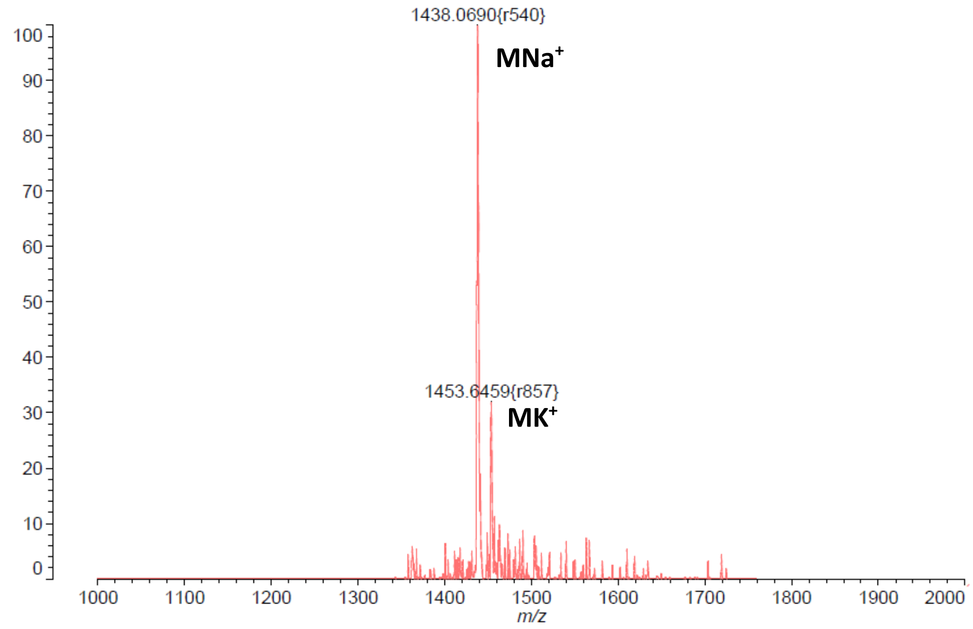

Figure S21. MALDI-TOS-MS spectra for purified $[WS]_4$.

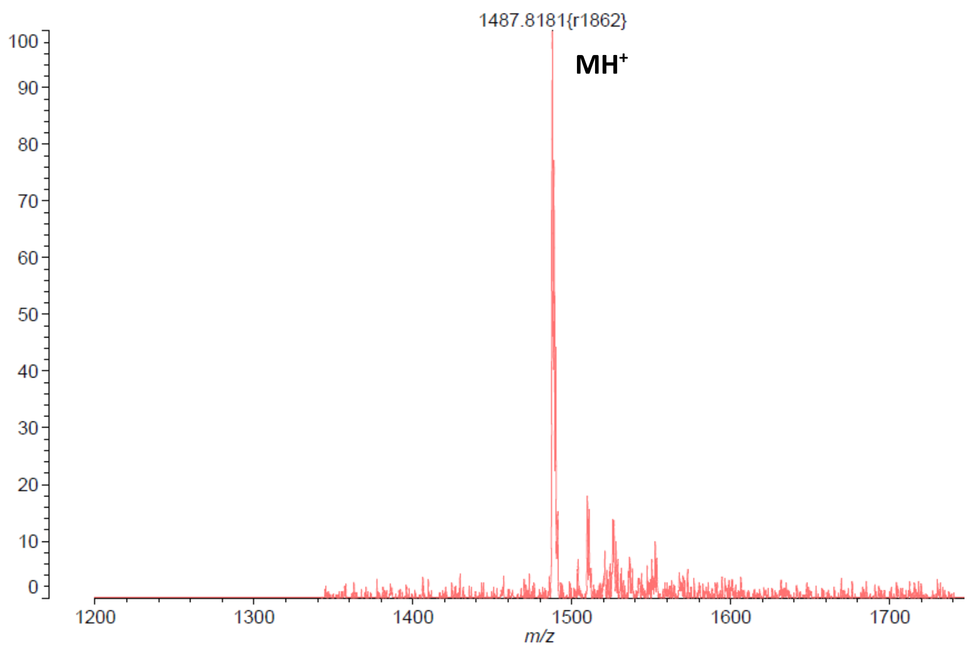

Figure S22. MALDI-TOS-MS spectra for purified $(WR)_4G$.

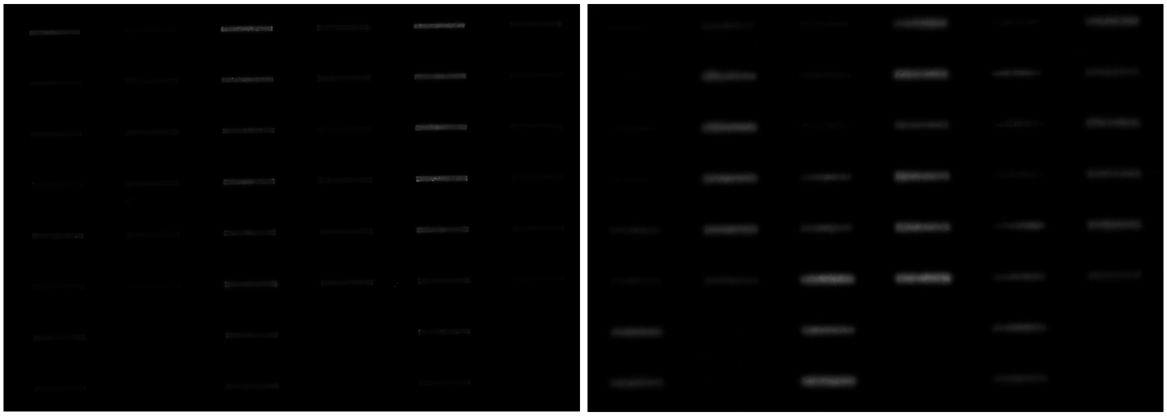

Figure S23. Nitrocellulose (left) and nylon (right) membranes for $[FKFE]_2$.

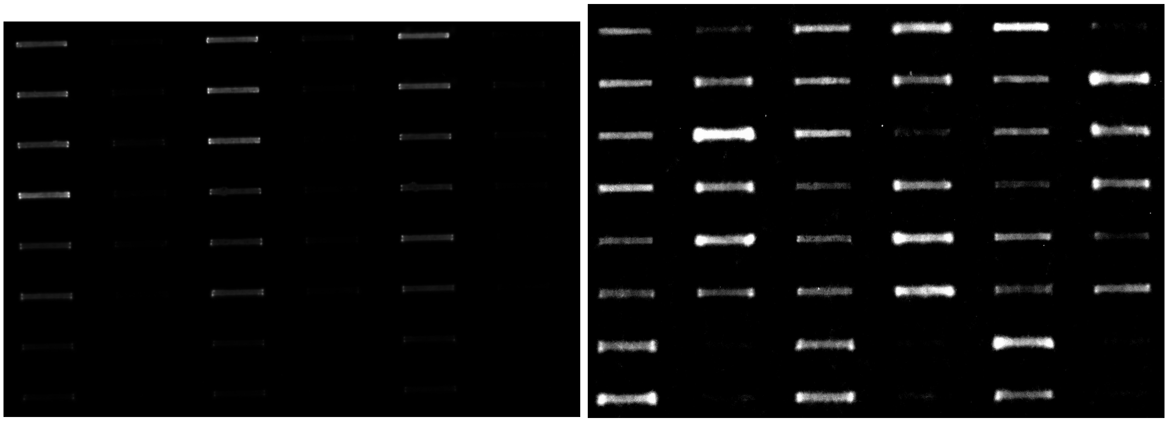

Figure S24. Nitrocellulose (left) and nylon (right) membranes for $[FK]_4$.

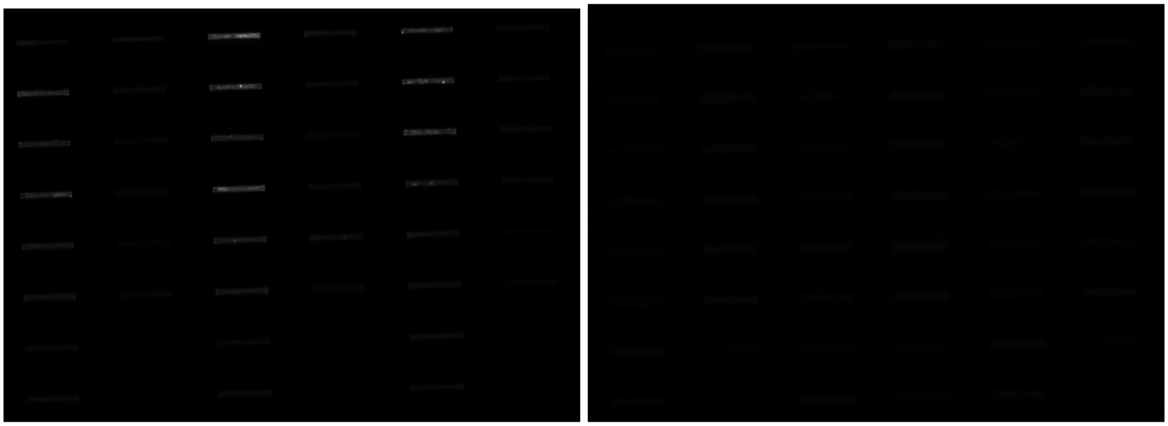

Figure S25. Nitrocellulose (left) and nylon (right) membranes for $[FR]_4$.

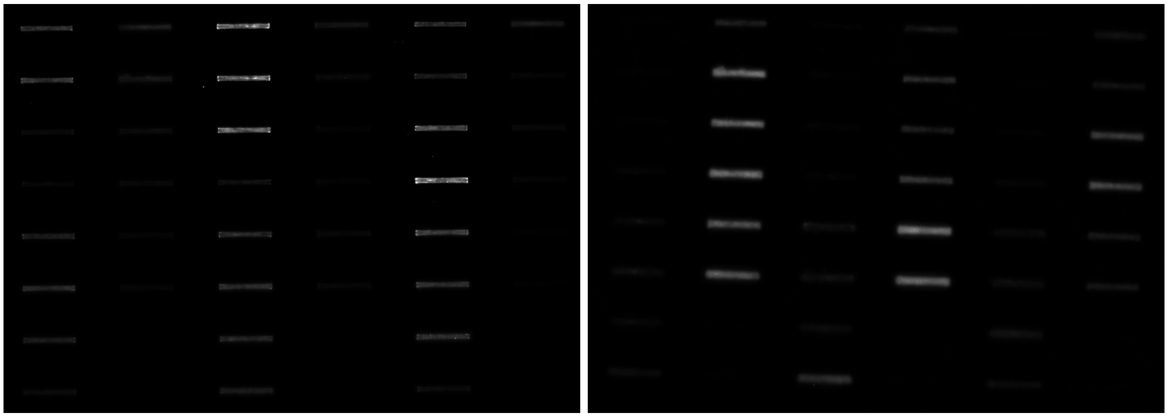

Figure S26. Nitrocellulose (left) and nylon (right) membranes for $[LR]_4$.

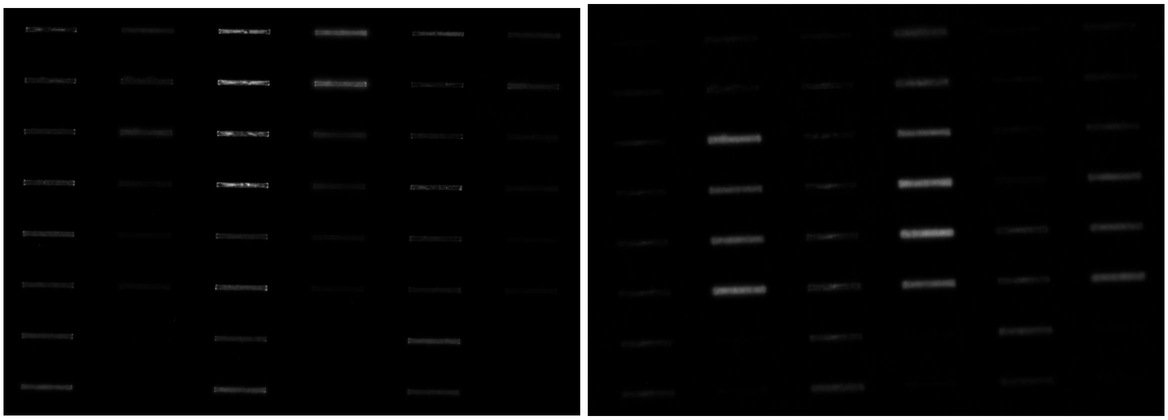

Figure S27. Nitrocellulose (left) and nylon (right) membranes for $[YR]_4$.

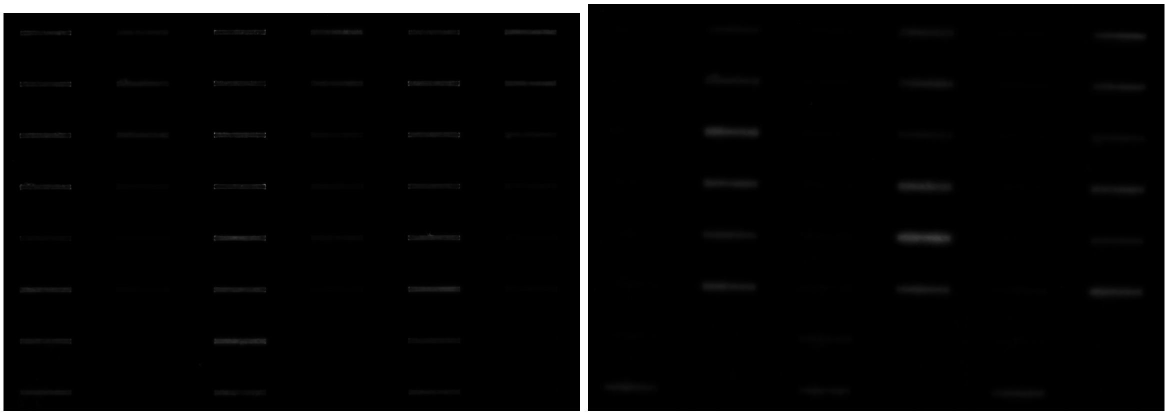

Figure S28. Nitrocellulose (left) and nylon (right) membranes for $[WR]_4$.

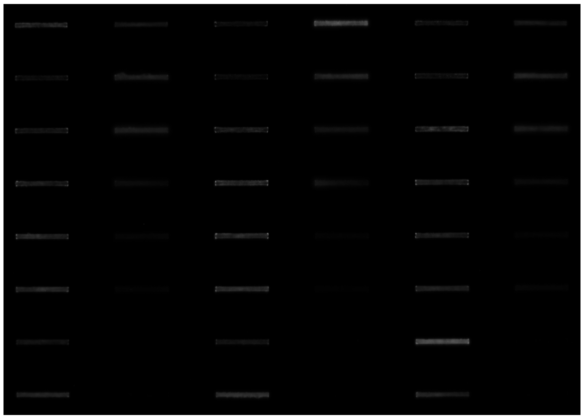
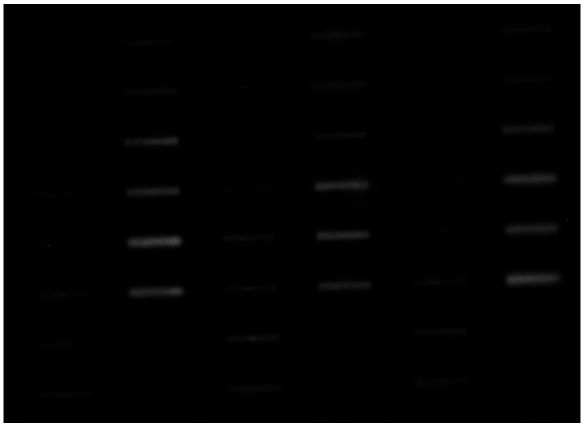

Figure S29. Nitrocellulose (left) and nylon (right) membranes for $[WK]_4$.

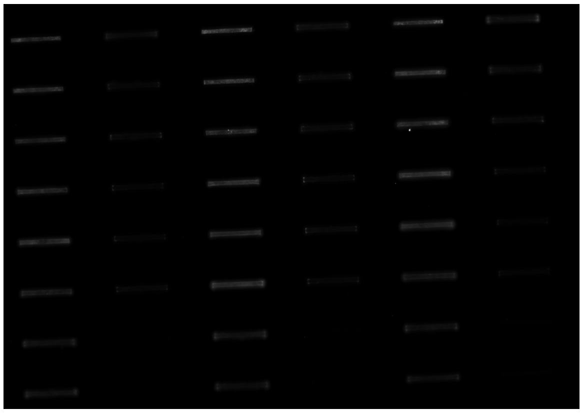
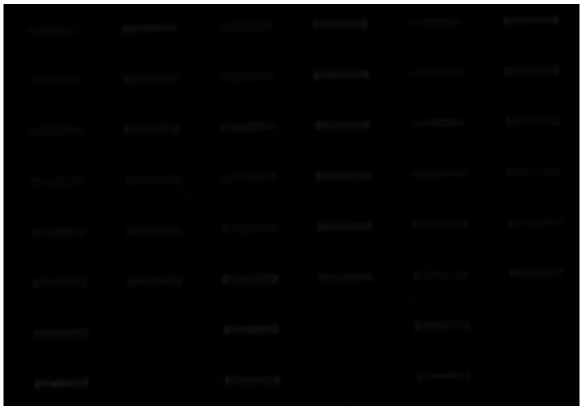

Figure S30. Nitrocellulose (left) and nylon (right) membranes for $[WH]_4$.

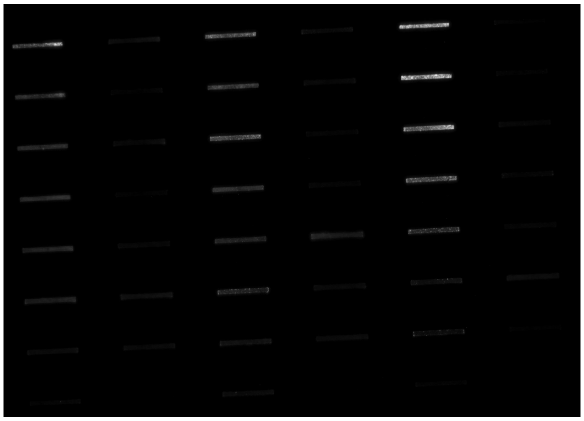
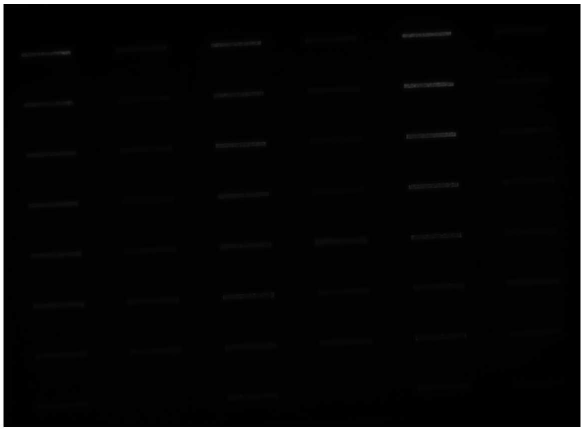

Figure S31. Nitrocellulose (left) and nylon (right) membranes for $[WS]_4$.

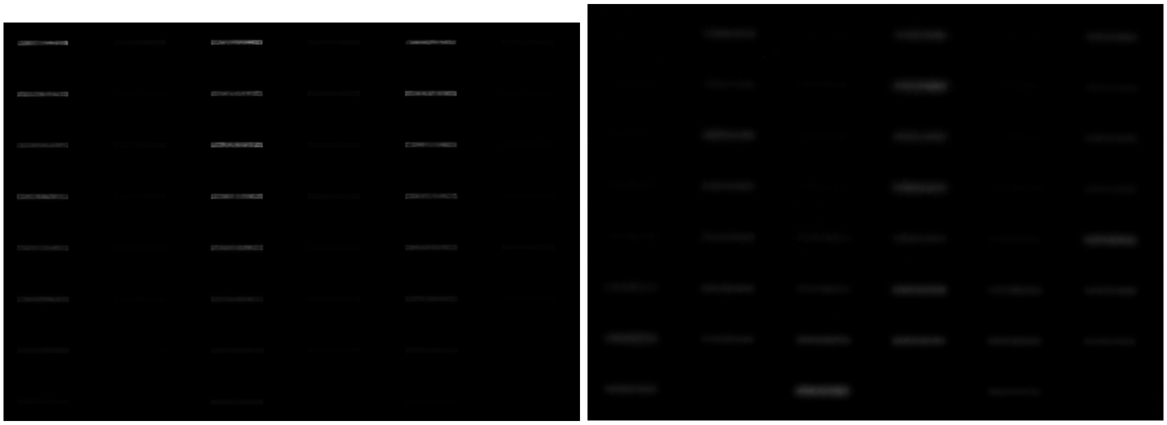

Figure S32. Nitrocellulose (left) and nylon (right) membranes for $(WR)_4G$.

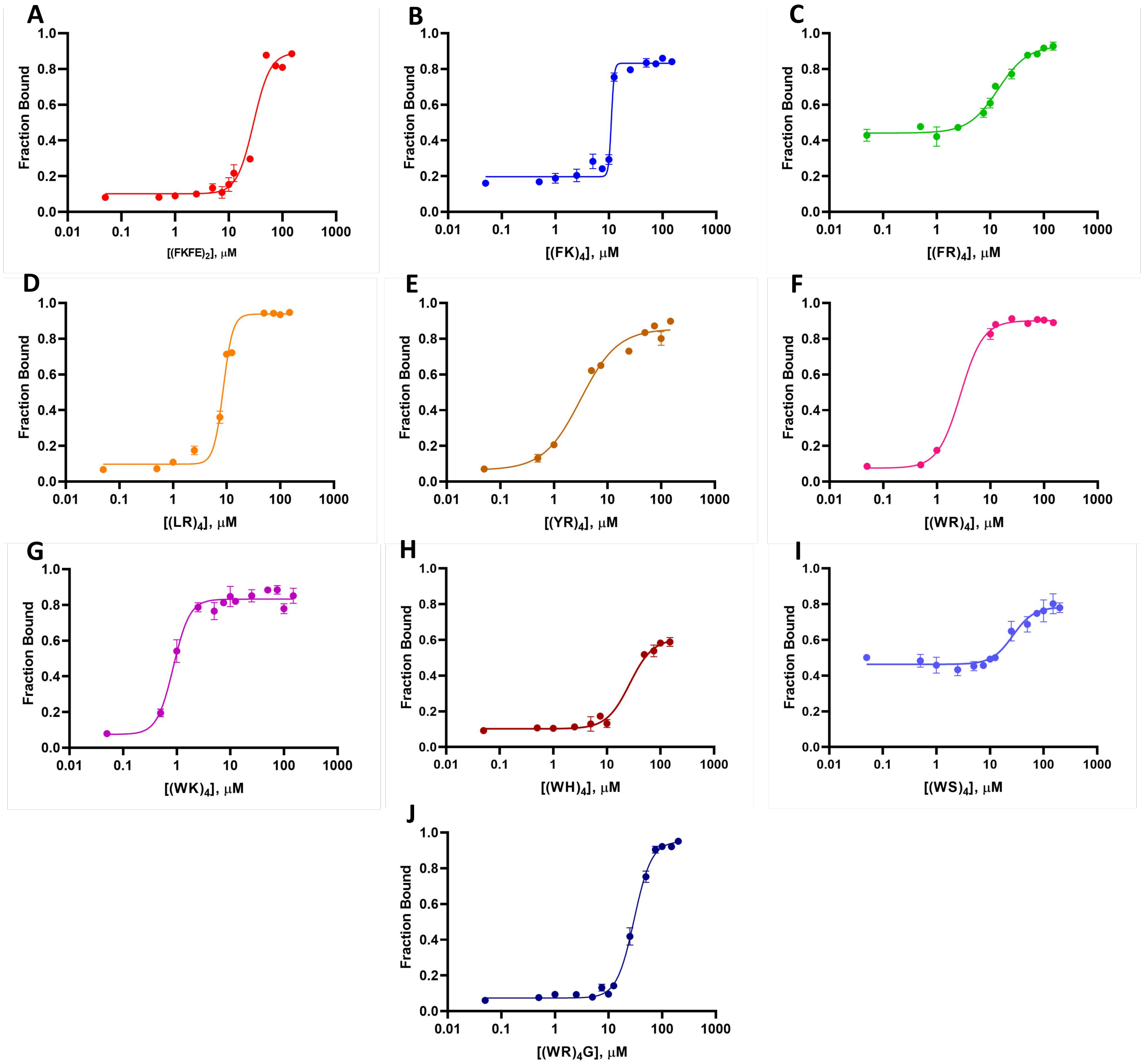

Figure S33. Plots of fraction bound (siRNA) vs. [CAP], (μM) obtained from slot blot filtration assay. **(A)** $[FKFE]_2$, **(B)** $[FK]_4$, **(C)** $[FR]_4$, **(D)** $[LR]_4$, **(E)** $[YR]_4$, **(F)** $[WR]_4$, **(G)** $[WK]_4$, **(H)** $[WH]_4$, **(I)** $[WS]_4$, and **(J)** $(WR)_4G$.

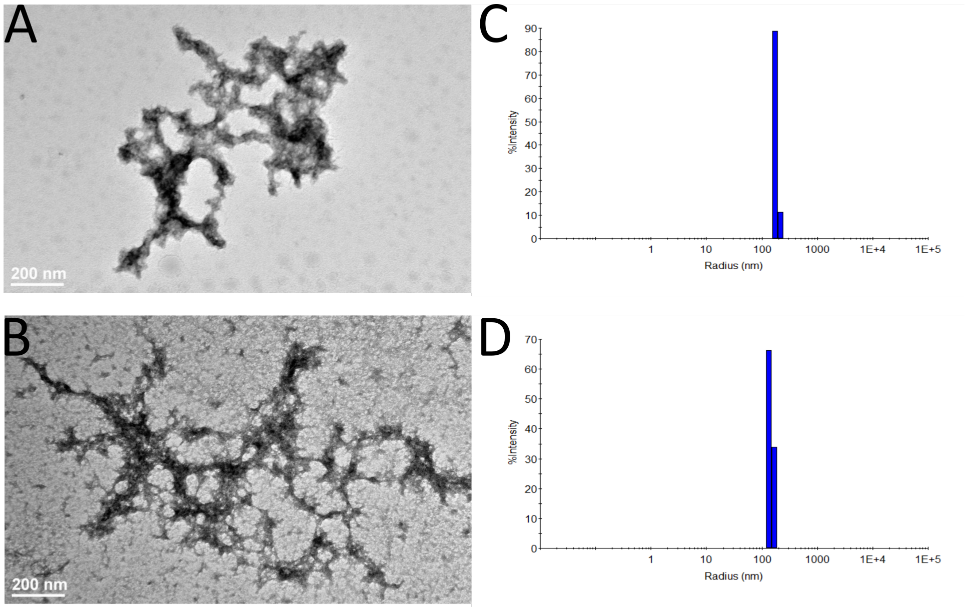

Figure S34. Nanoparticles characteristics of $[FKFE]_2$ complexed with siRNA. Transmission electron micrographs of **(A)** 1000-times peptide:siRNA excess and **(B)** 10-times peptide:siRNA excess. Dynamic light scattering plot in **(C)** 1000-times peptide:siRNA excess and **(D)** 10-times peptide:siRNA excess.

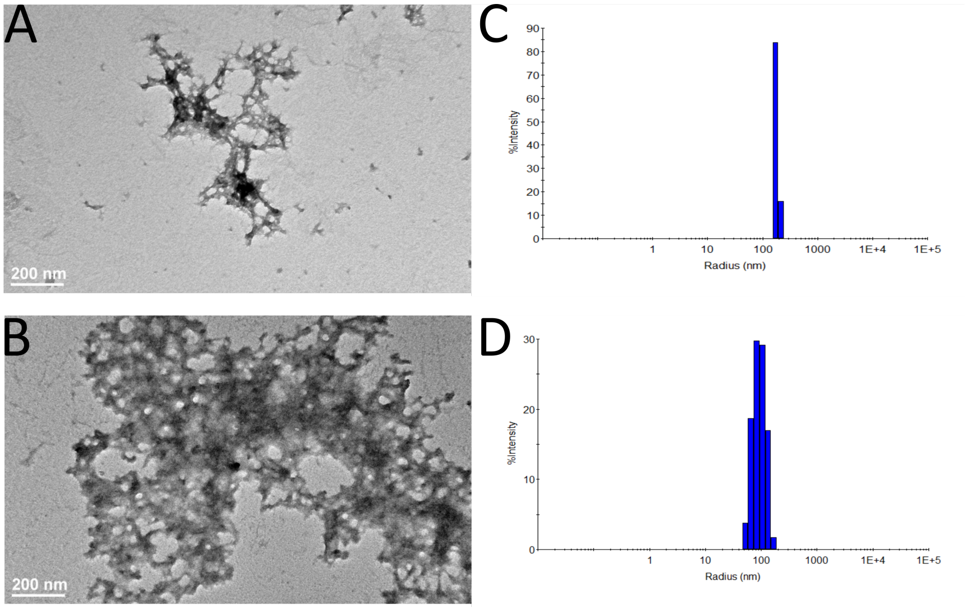

Figure S35. Nanoparticles characteristics of $[FK]_4$ complexed with siRNA. Transmission electron micrographs of **(A)** 1000-times peptide:siRNA excess and **(B)** 10-times peptide:siRNA excess. Dynamic light scattering plot in **(C)** 1000-times peptide:siRNA excess and **(D)** 10-times peptide:siRNA excess.

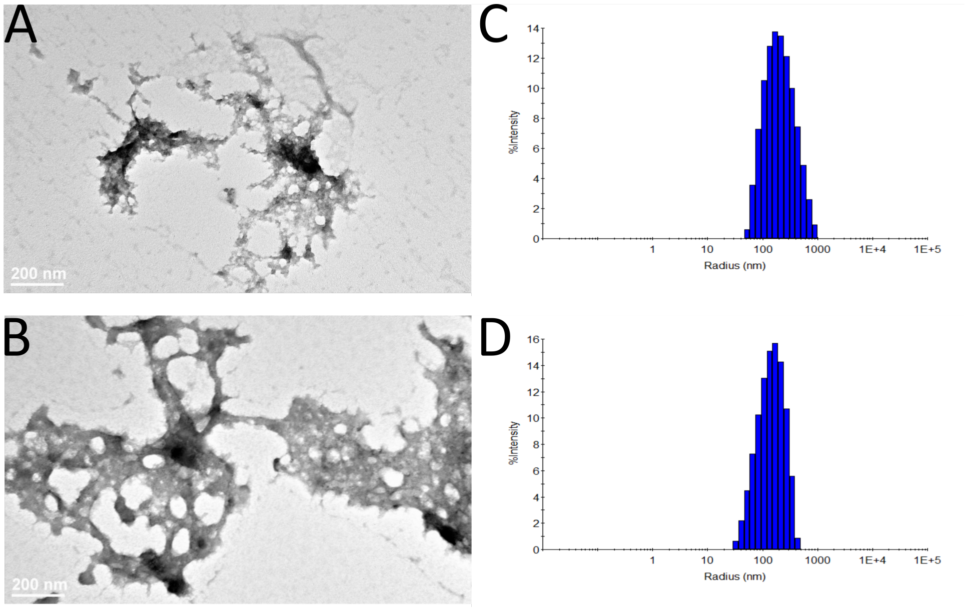

Figure S36. Nanoparticles characteristics of $[FR]_4$ complexed with siRNA. Transmission electron micrographs of **(A)** 1000-times peptide:siRNA excess and **(B)** 10-times peptide:siRNA excess. Dynamic light scattering plot in **(C)** 1000-times peptide:siRNA excess and **(D)** 10-times peptide:siRNA excess.

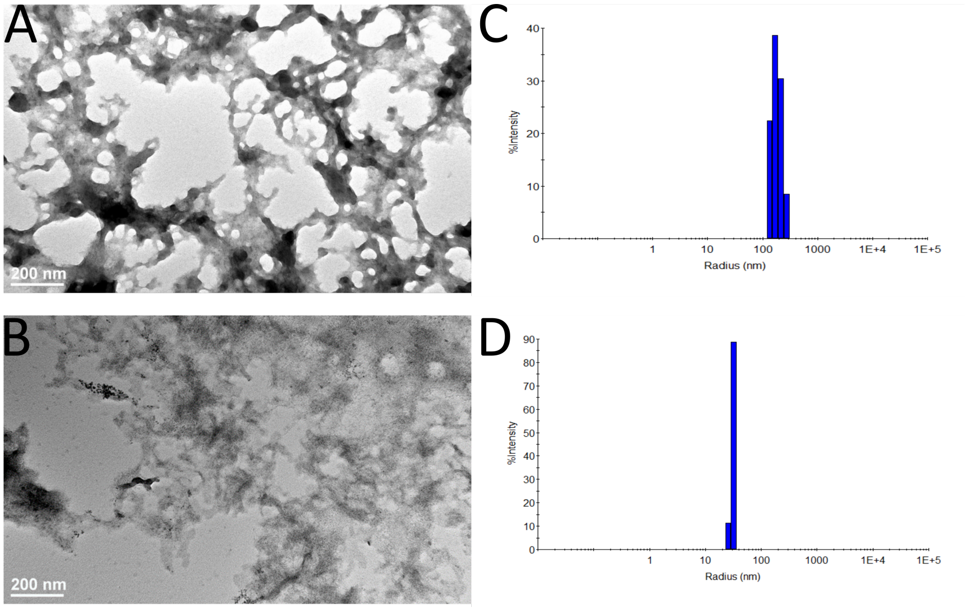

Figure S37. Nanoparticles characteristics of $[LR]_4$ complexed with siRNA. Transmission electron micrographs of **(A)** 1000-times peptide:siRNA excess and **(B)** 10-times peptide:siRNA excess. Dynamic light scattering plot in **(C)** 1000-times peptide:siRNA excess and **(D)** 10-times peptide:siRNA excess.

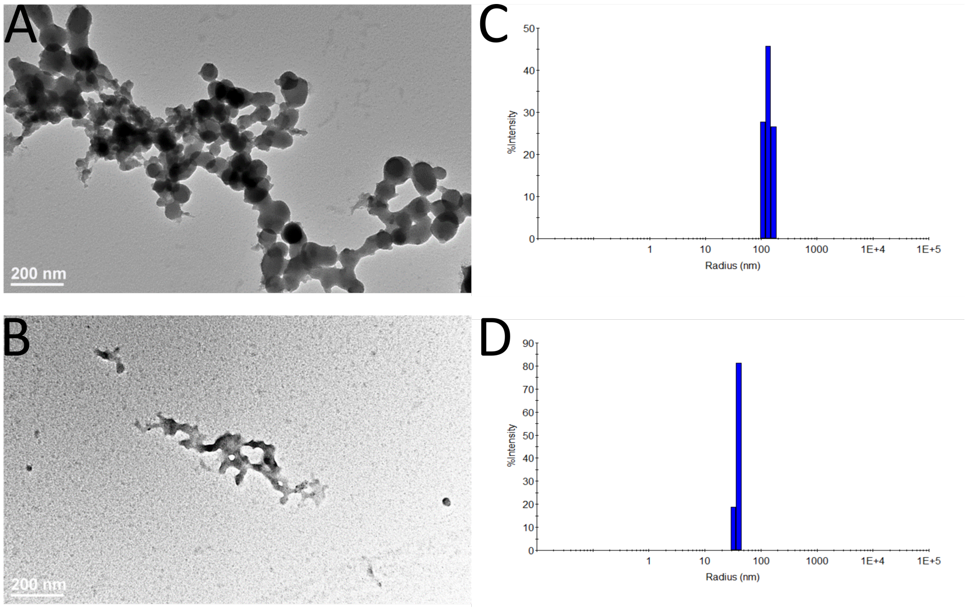

Figure S38. Nanoparticles characteristics of $[YR]_4$ complexed with siRNA. Transmission electron micrographs of **(A)** 1000-times peptide:siRNA excess and **(B)** 10-times peptide:siRNA excess. Dynamic light scattering plot in **(C)** 1000-times peptide:siRNA excess and **(D)** 10-times peptide:siRNA excess.

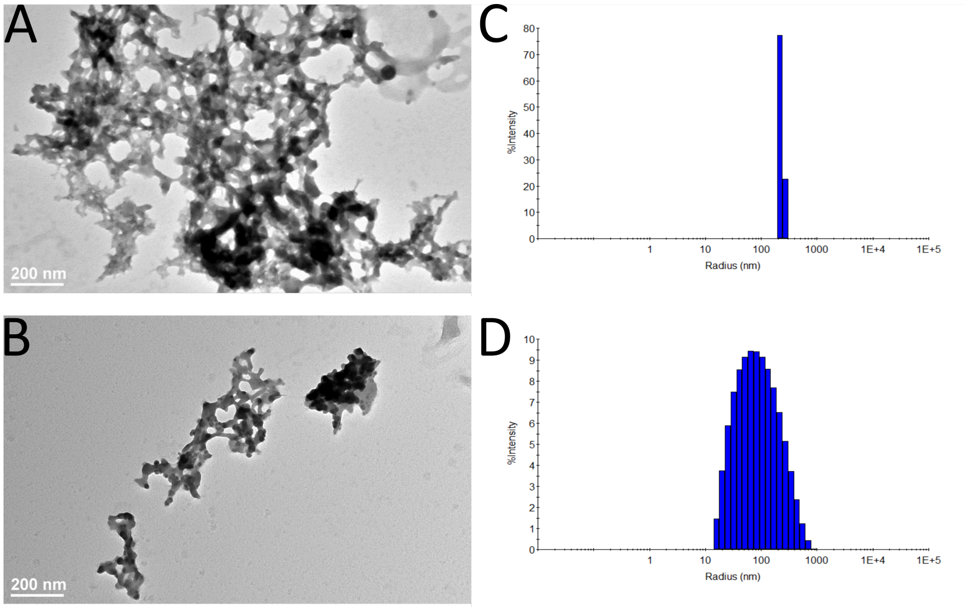

Figure S39. Nanoparticles characteristics of $[WR]_4$ complexed with siRNA. Transmission electron micrographs of **(A)** 1000-times peptide:siRNA excess and **(B)** 10-times peptide:siRNA excess. Dynamic light scattering plot in **(C)** 1000-times peptide:siRNA excess and **(D)** 10-times peptide:siRNA excess.

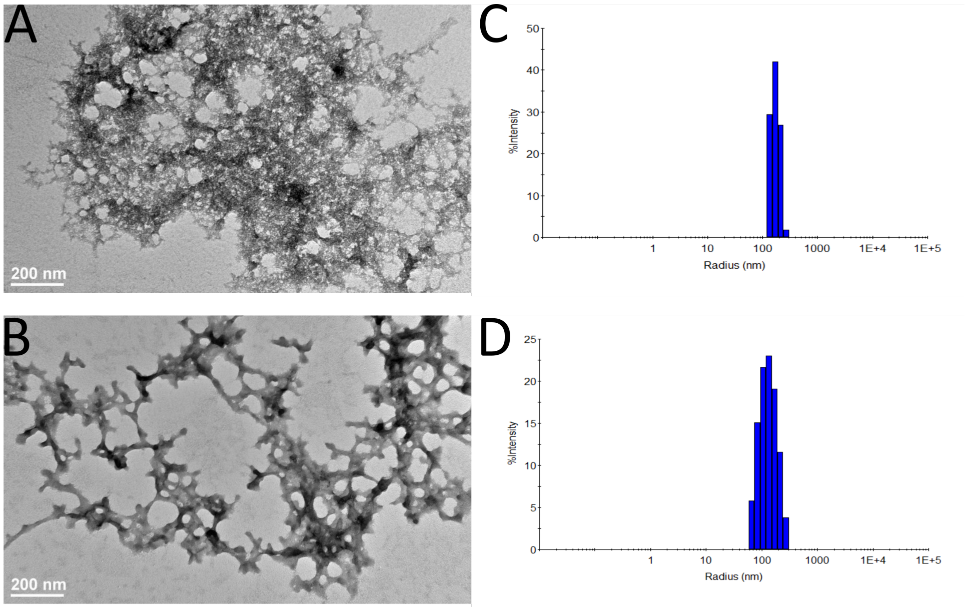

Figure S40. Nanoparticles characteristics of $[WK]_4$ complexed with siRNA. Transmission electron micrographs of **(A)** 1000-times peptide:siRNA excess and **(B)** 10-times peptide:siRNA excess. Dynamic light scattering plot in **(C)** 1000-times peptide:siRNA excess and **(D)** 10-times peptide:siRNA excess.

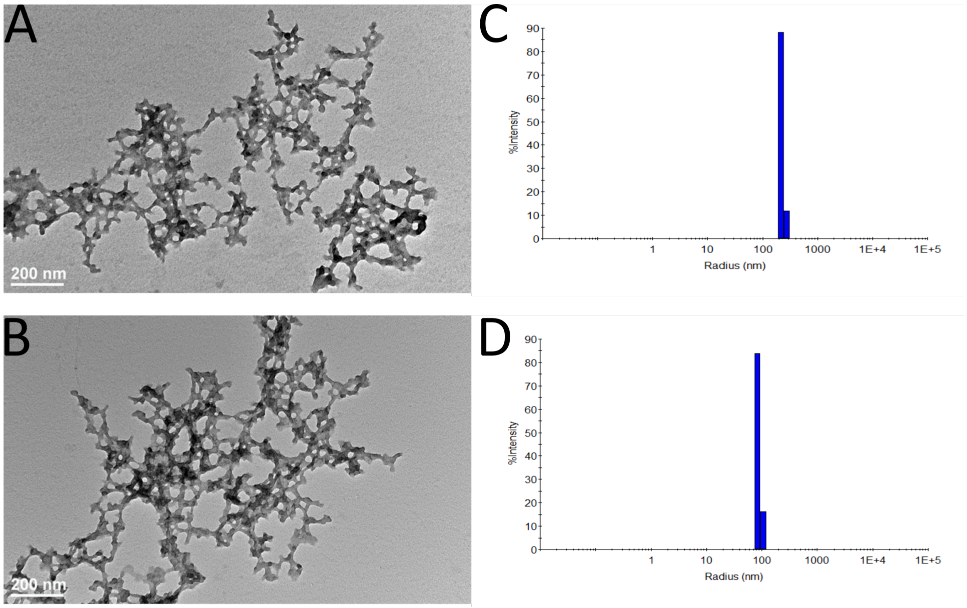

Figure S41. Nanoparticles characteristics of $[WH]_4$ complexed with siRNA. Transmission electron micrographs of **(A)** 1000-times peptide:siRNA excess and **(B)** 10-times peptide:siRNA excess. Dynamic light scattering plot in **(C)** 1000-times peptide:siRNA excess and **(D)** 10-times peptide:siRNA excess.

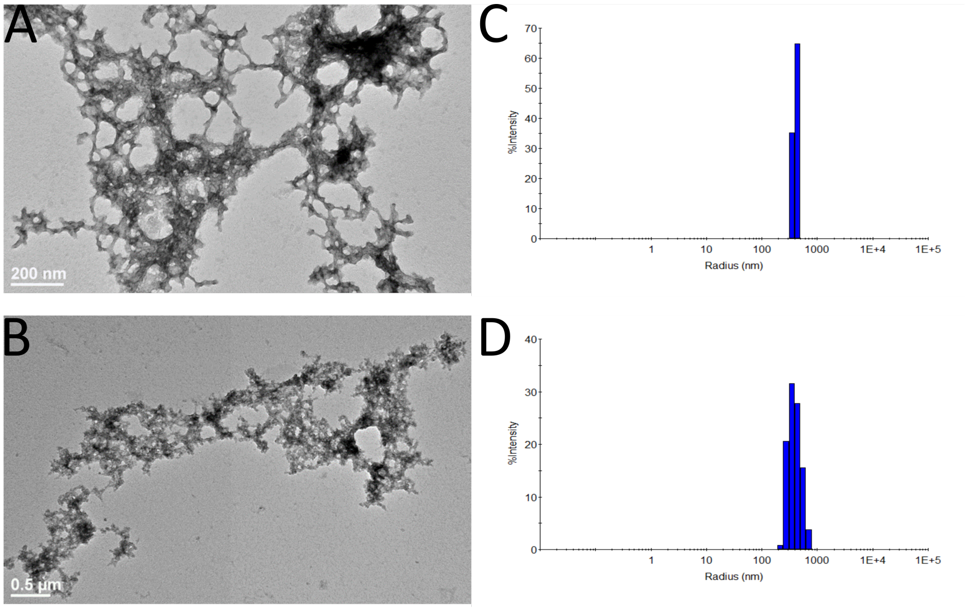

Figure S42. Nanoparticles characteristics of $[WS]_4$ complexed with siRNA. Transmission electron micrographs of **(A)** 1000-times peptide:siRNA excess and **(B)** 10-times peptide:siRNA excess. Dynamic light scattering plot in **(C)** 1000-times peptide:siRNA excess and **(D)** 10-times peptide:siRNA excess.

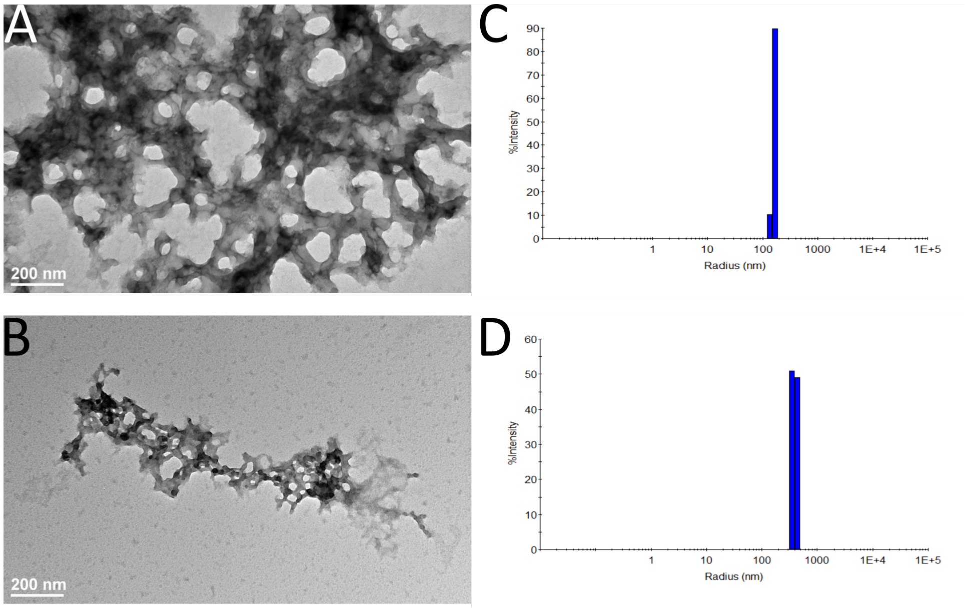

Figure S43. Nanoparticles characteristics of $(WR)_4G$ complexed with siRNA. Transmission electron micrographs of **(A)** 1000-times peptide:siRNA excess and **(B)** 10-times peptide:siRNA excess. Dynamic light scattering plot in **(C)** 1000-times peptide:siRNA excess and **(D)** 10-times peptide:siRNA excess.

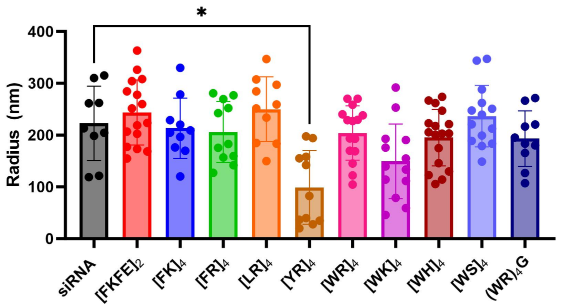

Figure S44. Radii of CAP-siRNA complexes at 1000-times peptide:siRNA excess. Statistical significance was evaluated using one-way ANOVA (n = 9, $*p \leq 0.05$).

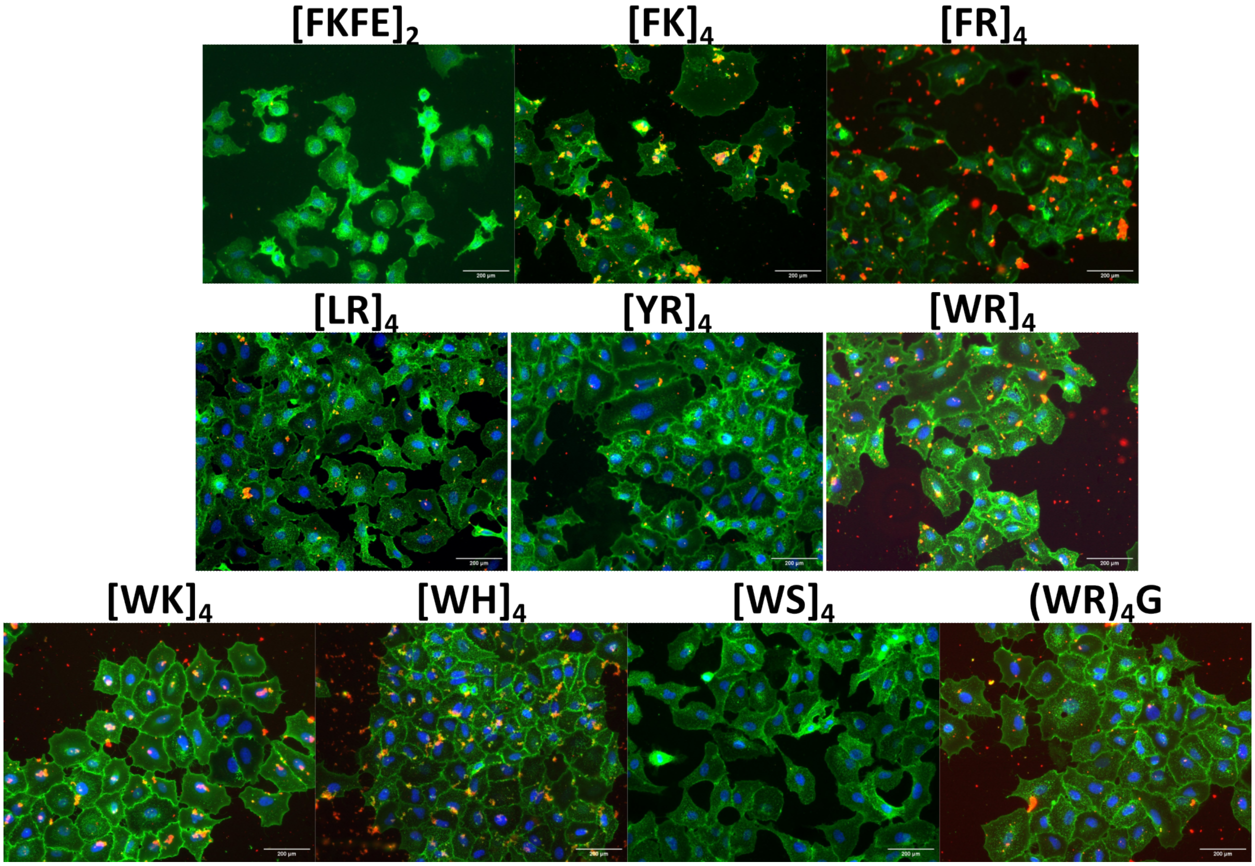

Figure S45. Delivery of fluorescently labelled siRNA (red) with CAPs in A549 lung adenocarcinoma cells. Nuclei are stained with DAPI (blue). Cell membranes are stained with Wheat Germ Agglutinin-Alexa 488 (green). CAP concentrations were 13.3 µM; siGlo Red-siRNA concentrations were 132 nM.

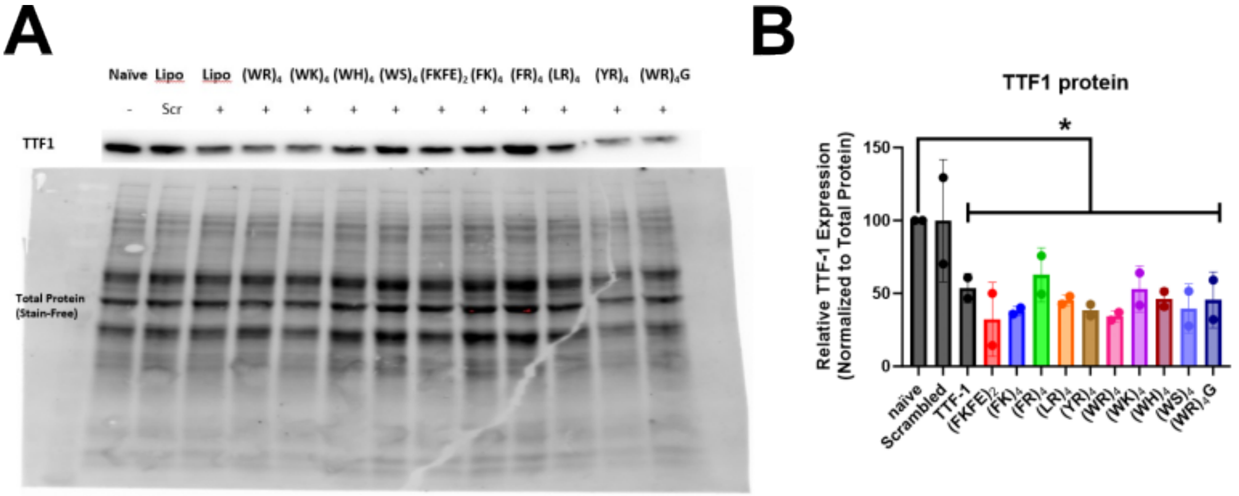

Figure S46. Knockdown efficiency of CAP-siRNA complexes against TTF-1 protein expression in A549 lung adenocarcinoma cells by Western Blot densitometry analysis**. (A)** Representative Western Blot TTF-1 protein expression against total protein in A549 lung adenocarcinoma cells exposed to no siRNA (naïve), lipofectamine-siRNA complexes, or CAP-siRNA complexes. **(B)** Relative TTF-1 protein expression as percent knockdown normalized to total protein determined by Western Blot densitometry analysis. Scrambled is control siRNA delivered with lipofectamine. TTF-1 is delivery of TTF-1 siRNA with lipofectamine (n = 2, *$p \leq 0.05$).

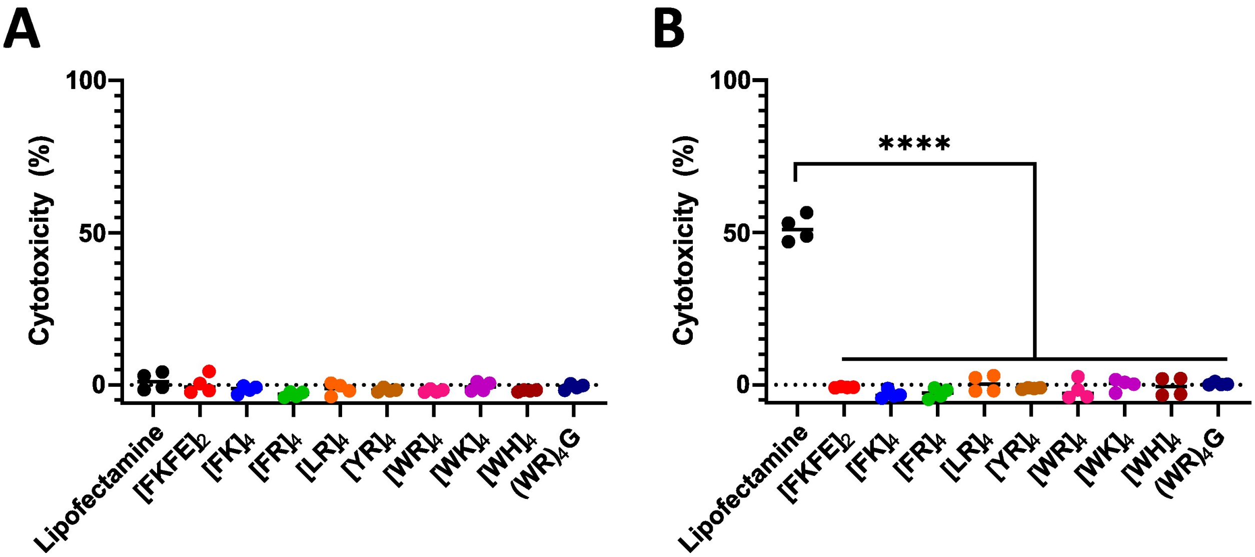

Figure S47. Cytotoxicity analysis of lipofectamine and CAPs (13.3 µM). **(A)** 4 h and **(B)** 24 h after dosing (n = 4, ****$p \leq 0.0001$).

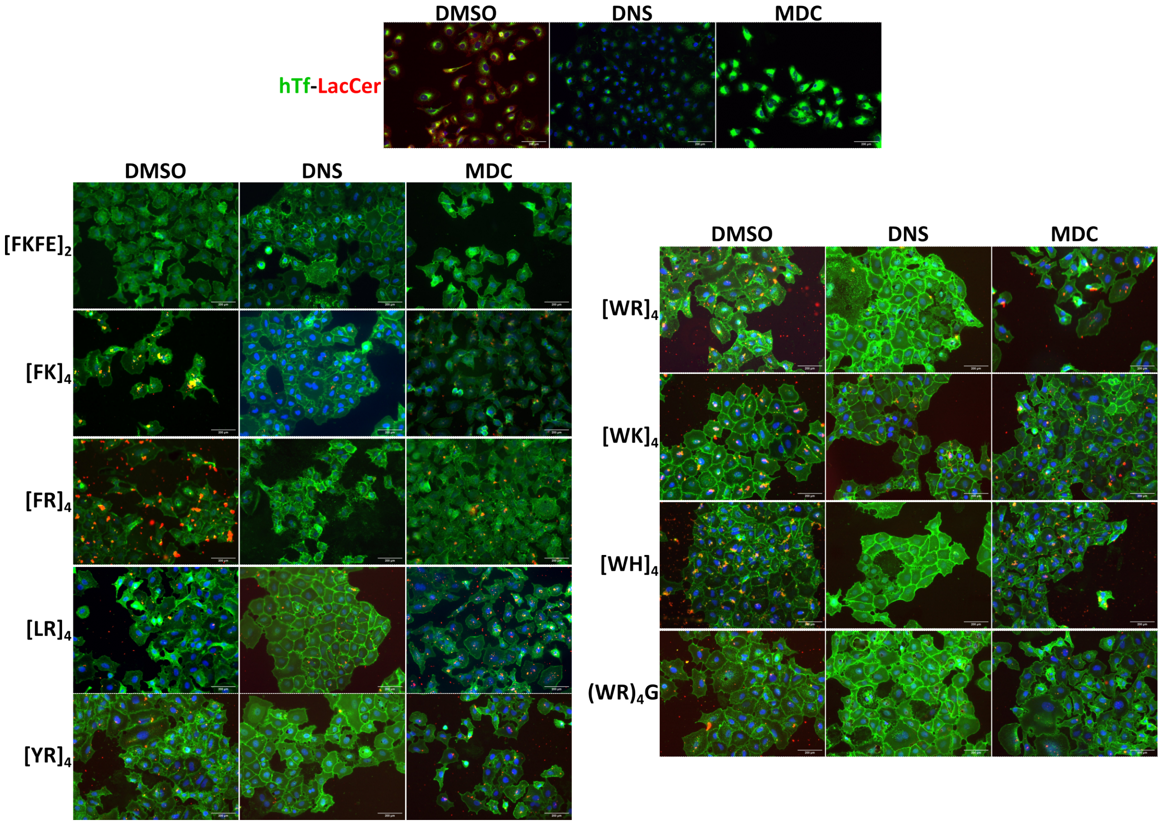

Figure S48. Images used to assess siRNA delivery efficiency of CAP-siRNA complexes in A549 lung adenocarcinoma cells in the presence of DNS (400 µM) or MDC (200 µM). siRNA is labelled with Cy3 (red). Nuclei are stained with DAPI (blue). Cell membranes are stained with Wheat Germ Agglutinin-Alexa 488 (green).

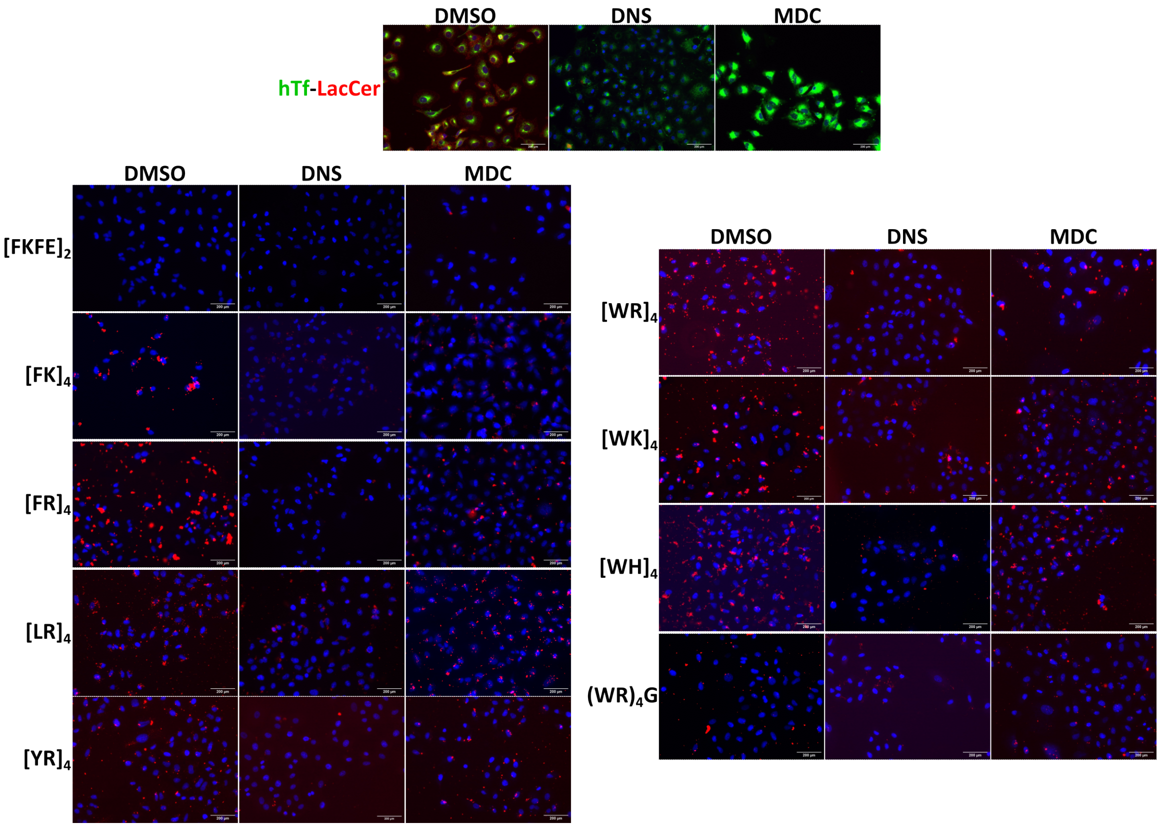

Figure S49. Images used to assess siRNA delivery efficiency of CAP-siRNA complexes in A549 lung adenocarcinoma cells in the presence of DNS (400 µM) or MDC (200 µM). siRNA is labelled with Cy3 (red). Nuclei are stained with DAPI (blue).

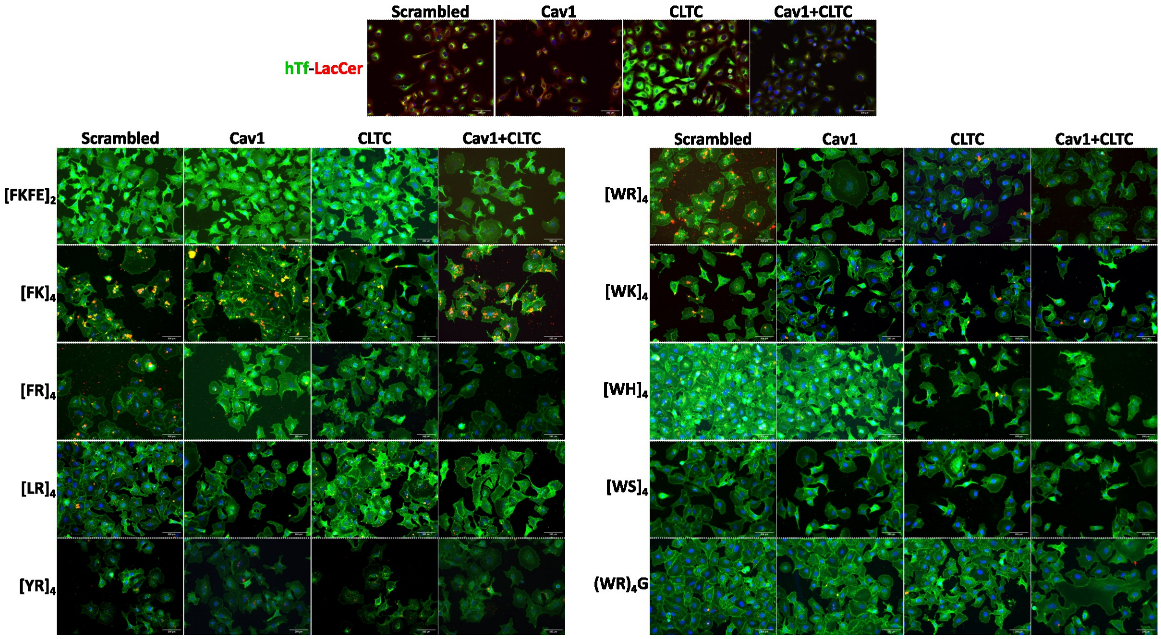

Figure S50. Delivery of CAP-siRNA complexes in Cav1$^+$/CLTC$^+$ (Scrambled), Cav1$^-$/CLTC$^+$ (Cav1), Cav1$^+$/CLTC$^-$ (CLTC), or Cav1$^-$/CLTC$^-$ (Cav1+CLTC) A549 adenocarcinoma cells. siRNA is labelled with Cy3 (red). Nuclei are stained with DAPI (blue). Cell membranes are stained with Wheat Germ Agglutinin-Alexa 488 (green).

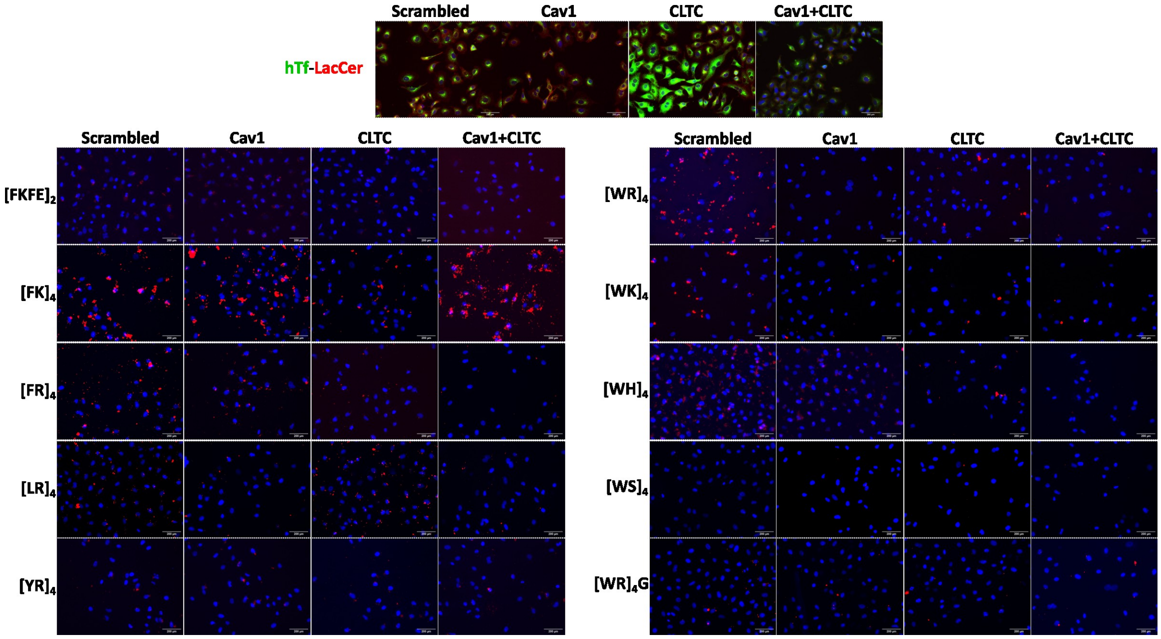

Figure S51. Delivery of CAP-siRNA complexes in Cav1$^+$/CLTC$^+$ (Scrambled), Cav1$^-$/CLTC$^+$ (Cav1), Cav1$^+$/CLTC$^-$ (CLTC), or Cav1$^-$/CLTC$^-$ (Cav1+CLTC) A549 adenocarcinoma cells. siRNA is labelled with Cy3 (red). Nuclei are stained with DAPI (blue).

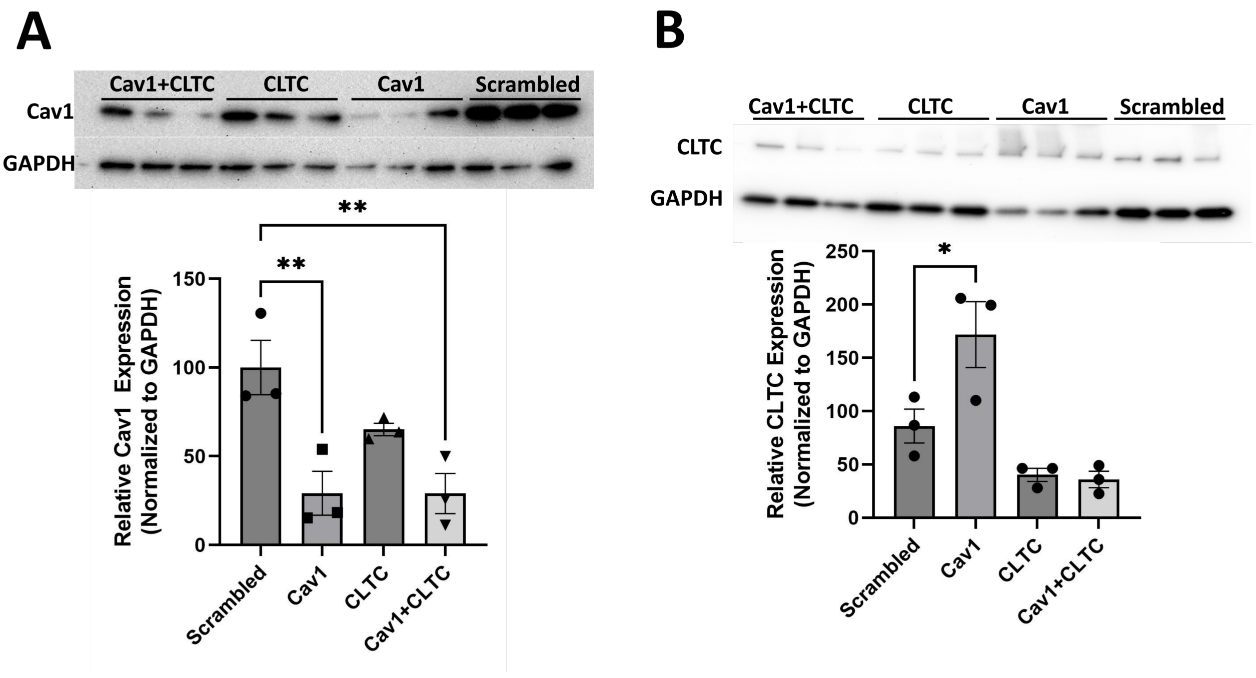

Figure S52. Western blot densitometry analysis verifying Cav1, CLTC, or Cav1+CLTC knockdown in A549 lung adenocarcinoma cells. **(A)** Relative Cav1 expression as percent knockdown normalized to GAPDH (n = 3, $**p \leq 0.01$). **(B)** Relative CLTC expression as percent knockdown normalized to GAPDH (n = 3, $*p \leq 0.05$).
